# Zinc Homeostasis in *Brucella ovis* Under Zinc Scarcity: Characterization of the ZnuABC Zinc Import System and of a Newly Identified Gene of the Zur Regulon

**DOI:** 10.3390/biology15191753

**Published:** 2026-10-02

**Authors:** Beatriz Tartilán-Choya, Ian S. Barton, R. Martin Roop, Nieves Vizcaíno

**Affiliations:** 1 Departamento de Microbiología y Genética, Universidad de Salamanca, 37007 Salamanca, Spain; beatarti@usal.es; 2 Department of Microbiology and Immunology, Brody School of Medicine, East Carolina University, Greenville, NC 27834, USA; bartoni19@ecu.edu (I.S.B.); roopr@ecu.edu (R.M.R.II); 3 Instituto de Investigación Biomédica de Salamanca (IBSAL), 37007 Salamanca, Spain

**Keywords:** *Brucella ovis*, zinc homeostasis, ZigA, ZnuA, ZntA, histidine, siderophore, TonB transport, virulence

## Abstract

The zinc import system ZnuABC is essential for the virulence of smooth (bearing O-polysaccharide chains) zoonotic *Brucella* species but remains uncharacterized in the non-zoonotic rough (lacking O-chains) species *Brucella ovis*, the causative agent of a type of ovine brucellosis of veterinary and economic importance. In this study, we constructed a panel of *B. ovis* mutants for genes of the *znuACB* locus that were characterized by evaluating growth, gene expression, protein abundance, metal content, and virulence in mice. We identified a ZigA ortholog (uncharacterized in the genus *Brucella* but linked to zinc homeostasis and histidine metabolism in other bacteria) that belongs to the Zur regulon and is transcriptionally modulated in a zinc-dependent manner as the ZnuABC system. A potential link of *B. ovis* ZigA with histidine catabolism and a crosstalk between the homeostasis of zinc and other metals have been identified as areas for further exploration. Remarkably, the *znuA*, *zigAznuA* and *znuACB* mutants of *B. ovis* displayed wild-type virulence in mice, which contrasts with the reported attenuation for the *znuA* mutant of smooth zoonotic *Brucella* species sharing a high degree of DNA homology. These results significantly enlarge the knowledge about the metallobiology of the genus *Brucella*, providing new perspectives for related research.

## Simple Summary

*Brucella ovis* causes non-zoonotic ovine brucellosis, a bacterial infection that is responsible for significant economic losses worldwide. Zinc homeostasis is an essential mechanism for pathogenic bacteria that must regulate zinc uptake and efflux to maintain the required levels for survival and to avoid the toxicity of zinc excess. Although a previous report evaluated zinc export in *B. ovis*, the mechanisms governing zinc uptake remained unknown. In this work, we constructed and characterized mutants for the zinc uptake system ZnuABC and for *zigA*, a gene uncharacterized in *Brucella* but associated in other bacteria with zinc homeostasis and histidine metabolism. We found that expression of these genes was regulated depending on zinc availability. A potential link between zinc and histidine homeostasis, as well as a crosstalk between the homeostasis of zinc and other metals, raised new directions for future research. Interestingly, removal of ZnuA, ZigAZnuA or ZnuABC did not impair the virulence of *B. ovis*, which contrasts with the reported attenuation for the ZnuA mutant of zoonotic *Brucella* species. Overall, these results provide new relevant insights about metal regulation in *Brucella*, which remains almost unexplored in *B. ovis*, and suggest new differences between zoonotic and non-zoonotic *Brucella* species, despite their high DNA homology.

## 1. Introduction

*Brucella ovis* is a Gram-negative bacterium belonging to the genus *Brucella* that causes a type of ovine brucellosis responsible for large economic losses in the livestock sector [1,2]. It is a non-zoonotic and rough species (lacking O-polysaccharide chains in the lipopolysaccharide [LPS]) that mainly produces epididymitis and other genital lesions in rams. This contrasts with smooth (bearing O-chains in the LPS) *Brucella melitensis* strains that have a high zoonotic potential and are highly abortifacient in sheep [3]. LPS O-polysaccharide chains are critical virulence determinants for smooth *Brucella* species (i.e., *B. melitensis*, *B. abortus* or *B. suis*, which preferentially infect sheep and goats, cattle and pigs, respectively) [4], but naturally rough *B. ovis* is virulent in sheep and in laboratory animals with its R-LPS being crucial for virulence [5].

*B. melitensis* Rev1 is a smooth attenuated vaccine that also confers cross-protection against rough *B. ovis*. However, it induces antibodies against O-polysaccharide chains that interfere with the serological diagnosis of infections caused by zoonotic smooth *Brucella* strains [1]. Consequently, its use is forbidden in *B. melitensis*-free areas, which results in an increase in *B. ovis* infection in these regions [2,6] and raises the need to develop a *B. ovis*-specific vaccine. Since vaccines commonly used in control and eradication campaigns targeting *B. melitensis* and *B. abortus* infections are homologous attenuated strains (*B. melitensis* Rev1 and *B abortus* S19 or RB51, respectively), a rough attenuated *B. ovis* strain would also be an interesting vaccine candidate against *B. ovis* infection in sheep. This approach requires a deeper understanding of the *B. ovis* virulence factors, which have been significantly less studied than those supporting the virulence of smooth *B. melitensis* and *B. abortus*.

Pathogenic smooth and rough *Brucella* strains are facultative intracellular bacteria able to establish a replicative niche inside phagocytes, a required characteristic to maintain a chronic infection [4,7]. Consequently, virulence attenuation is usually observed with *Brucella* spp. mutants exhibiting defective intracellular behavior. Metal homeostasis is crucial at the host–pathogen interface since metals are essential micronutrients for a wide variety of cellular processes but are toxic when present in excess [8,9,10]. Several mechanisms employed by the host to restrict metal availability or to intoxicate the bacterial pathogen, even in the intracellular environment, have been described. But bacteria have also developed strategies to overcome these challenges and maintain proper levels of metal ions [9,10].

Zinc ranks second in abundance among transition metal cofactors, following iron [11,12], and it is estimated that 5–6% of bacterial proteins are Zn-binding proteins intervening in essential metabolic pathways [13]. On the contrary, the Irving–Williams series, which categorizes first-row transition metals according to their relative competitiveness for ligand binding in a cellular environment, predicts high-affinity interactions of zinc with metal-binding sites [14]. Accordingly, zinc excess can affect metalloproteins that require metals other than zinc for their functionality, resulting in defective bacterial performance [11,12]. Vertebrate hosts possess specific transporters for zinc uptake and efflux that, along with additional mechanisms (e.g., calprotectin or metallothioneins functioning as extracellular and intracellular zinc-sequestering proteins), intervene in zinc intoxication or restriction processes affecting bacterial pathogens [10,12]. To counteract these defense strategies of the host, which are also detected in immune cells [10,12], bacteria have developed zinc homeostasis mechanisms generally relying on specific import and export systems that are switched on–off by transcriptional regulators able to sense zinc bioavailability inside the cell. However, other strategies, such as the presence of zinc metallochaperones that contribute to intracellular zinc mobilization–allocation, or the increase in proteins that do not require zinc and that are able to perform the same function as zinc-dependent proteins, have also been described [12,13,15].

The tripartite high-affinity Zn^2+^ uptake system ZnuABC is an ATP-binding cassette (ABC) transporter consisting of the periplasmic zinc-binding ZnuA protein, the ZnuB permease that spans the cytoplasmic membrane, and the cytoplasmic ATPase ZnuC [13,16]. This system, whose expression is controlled by the metalloregulator Zur in a zinc-dependent manner, together with the zinc exporter ZntA, which is transcriptionally regulated by another zinc-sensing protein called ZntR, have been studied in many bacteria as the main contributors to zinc uptake and efflux, respectively [13,16]. Extensive in vitro characterization has been conducted to understand their structure and function in bacterial zinc homeostasis, but reports evaluating their role in the interaction with the host are comparatively limited. Nevertheless, the involvement of the ZnuABC uptake system in virulence has been reported for several bacteria [11,12], but its dispensability for other bacterial pathogens has also been described [17]. Similarly, the ∆*zntA* mutants of *Chromobacterium violaceum* or *Vibrio parahaemolyticus* were reported to be attenuated [18,19], while ∆*zntA* mutants of other bacteria exhibit full virulence [20,21,22].

In the genus *Brucella*, the ZnuABC uptake system has only been studied in smooth *B. abortus* and *B. melitensis* where ZnuA was shown to be essential for the virulence in cellular models and mice [23,24,25]. While this system remains unexplored in rough *B. ovis*, a previous study of our team reported relevant differences between this species and smooth *B. abortus* 2308 regarding the ZntA-ZntR system, which is involved in Zn^2+^ efflux under zinc excess conditions [21]. Specifically, the ∆*zntR* mutant of *B. abortus* 2308, defective in the MerR-type transcriptional regulator that modulates expression of the Zn^2+^ exporter ZntA, showed virulence attenuation, which was linked to an associated increased transcription of *zntA* [20]. On the contrary, a *B. ovis* PA ∆*zntR* mutant showed reduced *zntA* transcription and was not attenuated in the mouse model [21]. An in-phase deletion of 300 bp removing 100 amino acids of the N-terminal cytoplasmic region of ZntA that contains a potential Zn^2+^ binding site was also detected in *B. ovis* [21]. However, the *B. ovis* ZntA exporter displayed equivalent activity to that of the *B. abortus* protein in response to toxic zinc levels and was revealed as a potent cadmium exporter also contributing to cobalt, copper, and nickel detoxification [21], characteristics that had not been previously described for the ZntA protein of *B. abortus* 2308 [20].

Considering the differences found between smooth *B. abortus* and rough *B. ovis* with respect to the ZntR-ZntA system and zinc efflux, as well as the knowledge gap concerning zinc uptake systems in rough *Brucella* species, the main goal of this work was to construct and characterize a panel of single and multiple mutants targeting the *znuACBzur* zinc import system of *B. ovis* PA and an adjacent gene encoding a protein annotated in the *B. ovis* genome as a zinc metallochaperone GTPase called ZigA. This gene has never been studied in the genus *Brucella*, and its characterization is a matter of interest considering that ZigA of *Acinetobacter baumannii* intervenes in zinc release from the histidine–zinc labile pool and is required for growth during zinc starvation and for virulence [26]. Moreover, the *B. ovis* genome was scanned searching for orthologs of additional genes associated with zinc homeostasis in other bacteria, and these genes were included in gene expression studies. The characterization of the thirteen genetically modified strains obtained in this work, including virulence assessment in the mouse model, offers new insights into the poorly studied zinc homeostasis in the genus *Brucella* and suggests new relevant differences with smooth *Brucella* species.

## 2. Materials and Methods

### 2.1. Cloning Vectors, Bacterial Strains and Culture Conditions

**Figure 1:**
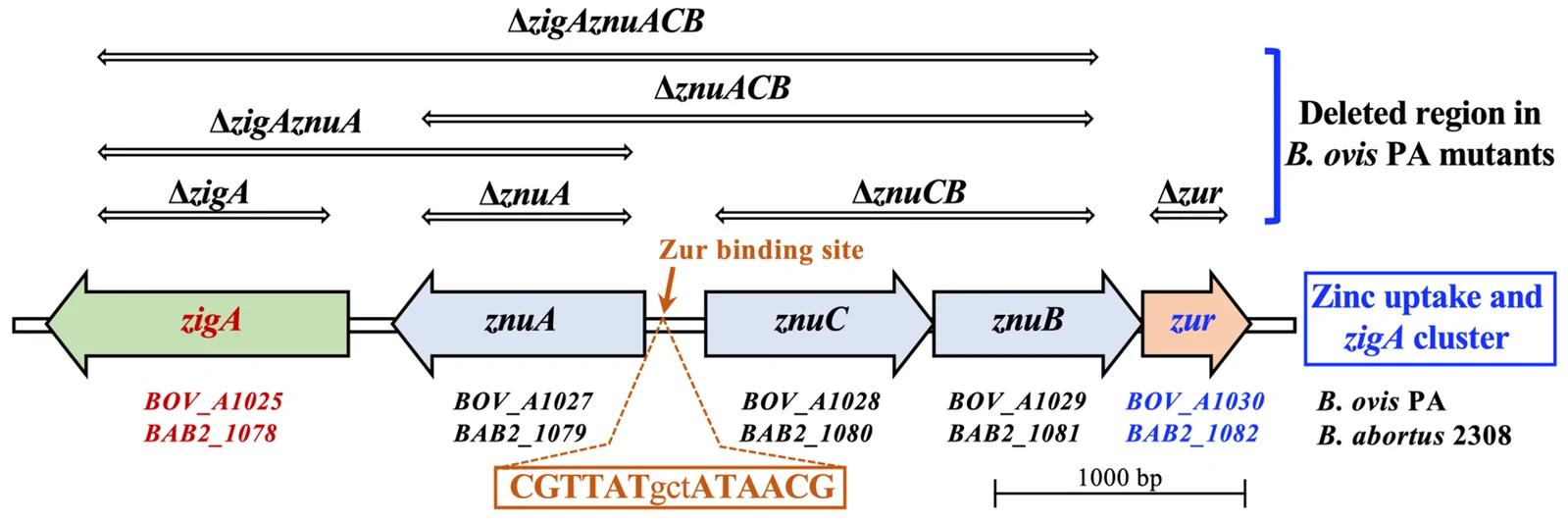
Genetic organization of the *znuACB-zur* zinc uptake cluster, and adjacent *zigA*, in *B. ovis* PA and *B. abortus* 2308. The predicted DNA binding site of the Zur transcriptional regulator, with the palindromic sequence of consensus Zur-box in other bacteria [29,30], is marked. DNA deletions in each constructed mutant are indicated by two-point arrows. A Δ*zntA*Δ*zur* double mutant was also constructed by deleting *zur* in a Δ*zntA* mutant, which is defective in a P_1B_-type ATPase involved in Zn^2+^ efflux [21].

Plasmid pCR^®^-Blunt (Life Technologies, Carlsbad, CA, USA) or pGEM^®^-T Easy (Promega, Madison, WI, USA) were used to clone PCR-amplified fragments. To obtain the *B. ovis* deletion mutants, target genes (Figure 1) were inactivated by overlapping PCR and cloned into pCVD-KanD, a plasmid that does not replicate in *Brucella* spp. and confers sucrose sensitivity and kanamycin resistance when integrated in the genome [27]. For complementation of mutant strains, wild-type genes were cloned into pBBR1MCS4, a plasmid that replicates in *Brucella* spp. and confers carbenicillin resistance [28].

Plasmids were transformed into and maintained in *Escherichia coli* strains CC118, JM109 or NZY5α [21] that were cultured on Luria Bertani medium, supplemented with 50 µg/mL kanamycin or carbenicillin when required, and incubated at 37 °C. *B. ovis* PA was used as the parental strain for the construction of the genetically modified strains listed in Table 1. *B. ovis* strains were cultured at 37 °C under a 5% CO_2_ atmosphere in trypticase soy broth or agar (TSB or TSA; Pronadisa-Laboratorios Conda, Torrejón de Ardoz, Spain) supplemented with 0.3% yeast extract (YE; Pronadisa-Laboratorios Conda, Torrejón de Ardoz, Spain) and 5% horse serum (HS; Gibco-Life Technologies, Grand Island, NY, USA). TSA-YE-HS or TSB-YE-HS culture media were supplemented with 5% sucrose or 50 µg/mL kanamycin or carbenicillin when required.

Growth curves of *B. ovis* PA-derived strains were established in TSB-YE-HS, supplemented with 50 µg/mL carbenicillin for strains containing pBBR1MCS4 or its recombinant derivatives, with bacterial cultures starting at optical density values at 600 nm (OD_600_) of 0.05. Incubation was performed at 37 °C with agitation at 120 rpm and under a 5% CO_2_ atmosphere. OD_600_ values were recorded for 3 days at several time points. Results presented are representative of three independent experiments. For procedures described below involving RNA extraction or proteomic and metal concentration evaluation, samples were collected and processed after 16 h of incubation (exponential growth phase). Since a minimal medium for *B. ovis* strains has not been developed, zinc deprivation conditions were established by incubation in the presence of 35 µM N,N,N′,N′-Tetrakis(2-pyridylmethyl)ethylenediamine (TPEN; Sigma-Aldrich, St. Louis, MO, USA), which is widely used as a Zn^2+^ chelator, or 0.5 mM of the more general metal chelator ethylenediaminetetraacetic acid (EDTA; Sigma-Aldrich, St. Louis, MO, USA). For zinc toxicity assays, ZnCl_2_ (Sigma-Aldrich, St. Louis, MO, USA) concentrations of 0.5, 3, or 5 mM were used, depending on the assay and strain analyzed. For gene expression analysis, 0.15 mM CdCl_2_ was added to the culture medium in some assays. Appropriate concentrations of these compounds (those slightly inhibiting growth of parental *B. ovis* PA or selected mutant strains) were previously determined [21] or established in this work (see below).

### 2.2. Primers, DNA Techniques and Analysis of Nucleotide and Protein Sequences

Primers used in this work, detailed in Supplementary Table S1, were purchased from IDT (Leuven, Belgium) and designed based on the published sequences of chromosome I and II (GenBank accession numbers NC_009505 and NC_009504, respectively) of *B. ovis* 63/290 (ATCC 25840) [31]. The published whole genome sequences of *B. abortus* 2308 (GenBank accession numbers AM040264 and AM040265) were also used to retrieve sequences of selected genes.

High-fidelity PCR reactions were performed with the NZYProof 2x Green Master Mix (NZYTech Lda., Lisboa, Portugal) and used for PCR amplification of the DNA fragments to be cloned into pCR^®^-Blunt or pGEM^®^-T Easy or to sequence the *zigA-zur* locus of *B. ovis* PA. Routine PCR reactions, employed for the verification of *B. ovis* recombinant strains, were done with the Red Taq DNA polymerase master mix (VWR, Leuven, Belgium). Sanger DNA sequencing, required for verification of plasmid constructs or to determine the nucleotide sequences of the *B. ovis* PA *zigA-zur* locus (Figure 1), was performed at the Nucleus Service of the University of Salamanca.

The KEGG database (Kyoto Encyclopedia of Genes and Genomes) was used to identify orthologs and paralogs of selected genes (https://www.kegg.jp/kegg/genes.html (accessed on 2 November 2025)). Protein subcellular localization and other protein characteristics were predicted with PSORTb v3.0.3 (Brinkman Laboratory, Simon Fraser University, Burnaby, BC, Canada) [32] and UniProt Knowledgebase [33] (https://www.uniprot.org (accessed on 7 March 2026)), respectively. The Basic Local Alignment Search Tool (BLAST^®^, blastn suite) was used to perform DNA similarity searches (https://blast.ncbi.nlm.nih.gov (accessed on 7 March 2026)) and EMBOSS Needle (European Bioinformatics Institute, Hinxton, UK) was used for pairwise sequence alignments of DNA or protein sequences (https://www.ebi.ac.uk/jdispatcher/psa/emboss_needle (accessed on 7 March 2026)). Protein stability was evaluated at https://web.expasy.org/protparam/ (accessed on 22 June 2026).

### 2.3. Construction of B. ovis Genetically Modified Strains

*B. ovis* mutant strains were obtained through a double homologous recombination process leading to the substitution of the wild-type gene by an inactivated gene resulting in an in-frame overlapping PCR that deletes almost the entire gene. The procedure was previously described [21,34] and involves a first stage where the 5′ and 3′ ends of each gene, together with 500–800 bp of adjacent DNA, are PCR-amplified with primers “target gene”MUT-F + “target gene”OVL-R and “target gene”OVL-F + “target gene”MUT-R, respectively. Both ends are then merged in a second PCR stage, with primers “target gene”MUT-F + “target gene”MUT-R, through the overlapping region of the two OVL primers used in the first PCR stage. PCR primers used in the construction of each mutant are described in (Supplementary Table S1). After cloning into pCR^®^-Blunt or pGEM^®^-T Easy and sequencing of the DNA insert, to discard undesired mutations, each inactivated gene was excised by digestion with restriction enzymes (*Xba*I plus *Sac*I or *Sph*I plus *Sal*I, depending on the gene) and cloned into pCVD-KanD to obtain the recombinant plasmids used to mutagenize *B. ovis*. Recombinant pCVD-KanD plasmids were introduced into *B. ovis* by electroporation and selection of intermediate, revertant and the desired mutant strains was performed, as described previously [21,34], according to their pattern of sucrose and/or kanamycin sensitivity/resistance and specific PCR reactions allowing their differentiation.

Recombinant pBBR1MCS4 plasmid bearing wild-type *zur* (pNV*zur*com2) was constructed by cloning the PCR fragment amplified with primers zurcom-F + znuCBMUT-R (Supplementary Table S1) first into pCR^®^-Blunt and then into pBBR1MCS4 after digestion with *Xba*I and *Sac*I restriction enzymes. This plasmid was selected and maintained in culture medium containing carbenicillin. Plasmids pNV*zntA*_PA_com5 and pNV*zntA*_2308_com5 were previously obtained [21] and were used either to complement the Δ*zntA*Δ*zur* mutant strains or to overexpress the cloned gene in selected strains previously obtained [21].

### 2.4. Relative Quantification of RNA Transcripts and End-Point RT-PCR

The procedure described previously was followed [21,35]. In brief, RNA was extracted, with the E.Z.N.A.^®^ Bacterial RNA Kit (Omega Bio-tek Inc., Norcross, GA, USA), from bacterial strains cultured for 16 h (exponential growth phase) in TSB-YE-HS, supplemented with 3 mM ZnCl_2_, 0.15 mM CdCl_2_, 35 µM TPEN or 0.5 mM EDTA where appropriate. After DNaseI treatment of RNA, cDNA was synthesized with the RevertAid H minus first strand cDNA synthesis kit (Thermo Scientific, Vilnius, Lithuania) and, as control of DNA absence, reactions lacking retrotranscriptase were also included. Real-time PCR was performed in a StepOnePlus™ apparatus (Applied Biosystems, Foster City, CA, USA) using NZYSpeedy qPCR Green Master Mix (2×), ROX plus (NZYTech Lda., Lisboa, Portugal). Primer pairs for each target gene are listed in Supplementary Table S1. Relative expression was evaluated with the 2^−ΔΔCt^ method using the StepOne™ Software v2.3. *B. ovis* PA cultured in TSB-YE-HS and 16S were used as the reference strain and gene, respectively [36]. Three technical replicates and three independent biological samples were used for each gene, strain, and culture condition. Results are expressed as mean ± SD of the log_2_ of the relative quantity (log_2_RQ), and ΔCt values were used for statistical analysis [37].

When necessary, cDNA was subjected to end-point PCR, and the amplified fragments were visualized on a 2% agarose gel after electrophoresis using an O’GeneRuler Low Range DNA ladder (Thermo Scientific, Vilnius, Lithuania) as a size marker.

### 2.5. Protein Techniques and Evaluation of Metal Concentration

Sodium dodecyl sulfate polyacrylamide gel electrophoresis (SDS-PAGE) [38] was performed, on bacterial cells grown in TSB-YE-HS for 16 h, using 14% acrylamide/bisacrylamide gels and a Protein II xi cell (Bio-Rad, Hercules, CA, USA). Pre-stained protein marker VI (Applichem-Panreac, Barcelona, Spain) was used for protein molecular weight standards and separated protein bands were stained with Coomassie blue. A Chemidoc MP apparatus (Bio-Rad) was used for image acquisition.

The proteomic analysis of SDS-PAGE-selected protein bands was performed, as described previously [21], in the proteomics facility of Centro de Investigación del Cáncer, Salamanca, Spain, according to its standardized procedures. In brief, protein bands excised from the gels were digested with trypsin and submitted to reversed-phase LC-MS/MS. Identification of proteins was performed by searching the MS/MS spectra against the *B. ovis* Uniprot proteome database using the Andromeda algorithm [39] and MAXQUANT [40]. The abundance of proteins in the selected gel slices was evaluated using the Intensity-Based Absolute Quantification (iBAQ) score [41]. Results are represented as means ± SD (*n* = 2) of log_10_ iBAQ scores in two SDS-PAGE gels.

For the determination of metal concentrations present in bacterial cells, strains were cultured on TSB-YE-HS for 16 h in the presence of 35 µM TPEN, 0.5 mM EDTA or 30 µM ZnCl_2_ when desired. A volume equivalent to 2 mL of an OD_600_ value of 1 was centrifuged and the sediment washed and resuspended in 1 mL of 0.9% NaCl. A volume of 100 µL was used to determine the protein concentration using a Bradford assay solution (Applichem GmbH, Darmstadt, Germany). The remaining 900 µL was centrifuged and sedimented bacteria were incubated at 80 °C for 1 h to kill the bacteria. Metal concentrations were determined, after resuspending the bacterial pellets in 10 mL of 1% HNO_3_, by inductively coupled plasma mass spectrometry (ICP-MS) with an Agilent 7800 apparatus (Agilent Technologies, Santa Clara, CA, USA) at the Nucleus Service of the University of Salamanca following its standardized procedures. Results are represented as means ± SD (*n* = 3) of ng metal/mg protein. Metal concentrations were also assessed in 1 mL TSB-YE-HS with 9 mL of 1% HNO_3_, and results are represented as means ± SD (*n* = 3) of ng metal/mL.

### 2.6. Virulence in the Mouse Model

To evaluate the virulence of the bacterial strains in mice, the procedure previously described was followed [42]. Briefly, 6-week-old BALB/c mice (Charles River Laboratories, Chatillon-sur-Chalaronne, France) were intraperitoneally inoculated with 10^6^ CFU of each bacterial strain. Bacterial counts in the spleen were determined at week 3 and 7 post-infection in five mice per group and time point. Results were expressed as means ± SD (*n* = 5) of log_10_ CFU/spleen values at each time point. The genotype of bacterial strains recovered from spleens was checked by PCR with specific primers for each deleted gene/s. The Spanish and European legislation for research with animals (RD 53/2013 and directive 2010/63/EU) was followed to design and perform experiments with mice.

### 2.7. Statistical Analysis

The GraphPad Prism Software version 7.0e (GraphPad Software Inc., San Diego, CA, USA) was used for statistical comparisons with one-way ANOVA, followed by Tukey’s test, or t-test for multiple or single comparisons, respectively. Statistically significant differences (*p* ≤ 0.05) were established with a 95% confidence interval.

## 3. Results

### 3.1. Sequence Analysis of the znuACBzur Locus of B. ovis PA and Search for Orthologs of Genes Related to Zinc Import in Other Bacteria

Analysis of the *znuACBzur* locus revealed that the protein encoded by *BOV_A1025*, the gene located at the 3′-end of *znuA* (Figure 1), is annotated in the *B. ovis* genome as zinc metallochaperone GTPase ZigA. The ZigA protein of *B. ovis* shares 56.4% amino acid identity with ZigA (Zur-induced GTPase A) of *Acinetobacter baumannii*, encoded by *A1S_3411*, and 70.6% identity with the YciC zinc chaperone of *Agrobacterium tumefaciens*, encoded by *Atu3181*. Both of the latter proteins have been shown to have an important role in survival under conditions of zinc deprivation [26,43]. Since this gene and its encoded protein have not been studied in the genus *Brucella*, *BOV_A1025* was also targeted for mutagenesis.

**Figure 2:**
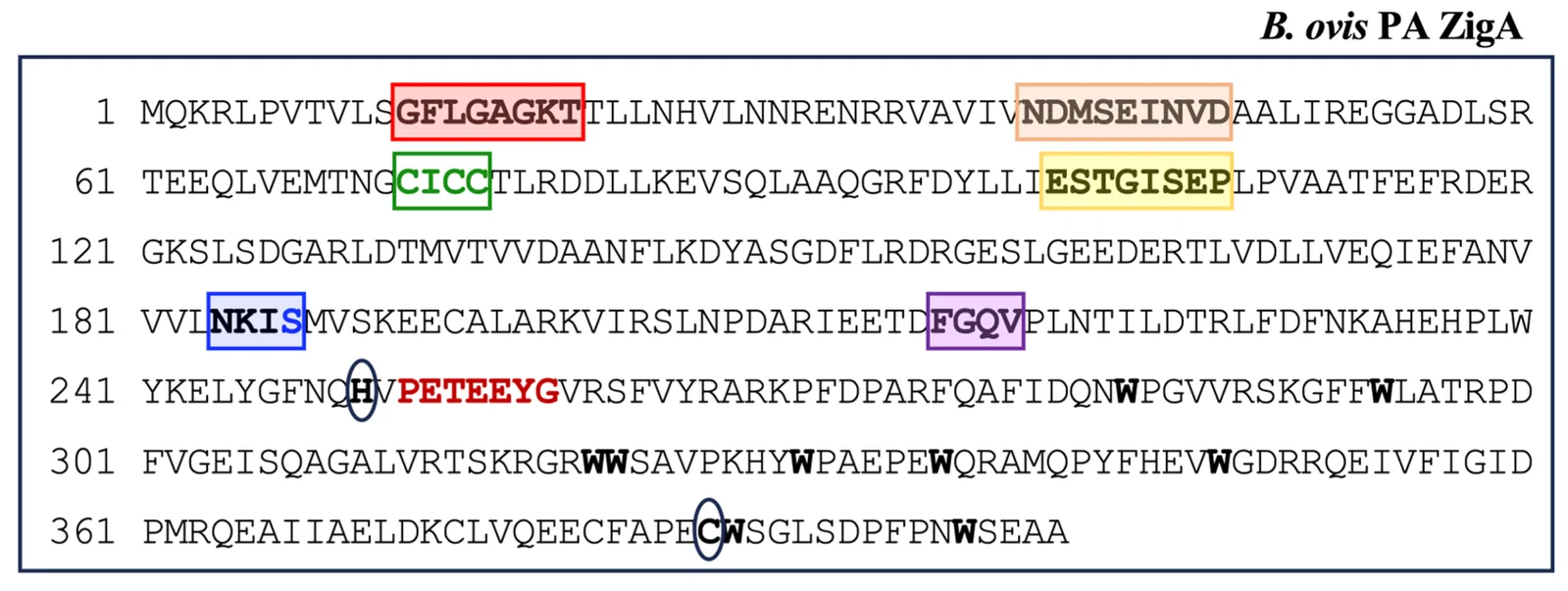
Amino acid sequence of the *B. ovis* PA ZigA protein highlighting its conserved motifs corresponding to G3E P-loop GTPases grouped in cluster 1 of COG0523 proteins [44]. Conserved motifs of the five G-loops of the N-terminal G-domain (responsible for GTPase activity) are in bold and framed as follows: G1/Walker A (red), G2 (orange), G3/Walker B (yellow), divergent G4 motif NKIS, instead the canonical NKXD sequence, (blue), and G5 (purple). The putative CXCC metal-binding motif located between G2 and G3 loops is framed in green. The bold red lettering represents the linker connecting the G-domain with the C-terminal domain, the latter involved in the recognition and release of client proteins to be metallated. The characteristic tryptophan residues of the C-terminal region are highlighted in bold and two conserved H and C residues [44] are marked with black circles.

Based on its amino acid sequence (Figure 2), ZigA of *B. ovis* PA is predicted to be a cytoplasmic protein (PSORTb v3.0.3) that contains the characteristic motifs described for G3E P-loop small GTPases belonging to the Cluster of Orthologous Groups 0523 (COG0523), specifically for those grouped in cluster 1 [44]. This cluster mainly consists of bacterial GTPases regulated by Zur such as *A. baumannii* ZigA [44]. This protein has been described as a metallochaperone transferring zinc to acceptor enzymes intervening in the catabolic pathway of histidine under zinc-limiting conditions [26]. COG0523 proteins are structurally composed of two domains, with characteristic motifs, that are also present in the *B. ovis* ZigA ortholog (Figure 2): (i) the N-terminal G-domain, of about 250 amino acids, contains the GTPase domain, which is composed of five G (GTP-binding) loops and is responsible for metal interaction as well as for GTP binding and hydrolysis [44,45], and (ii) the C-terminal domain that displays more variability, although it contains several conserved tryptophan residues [26,44], and is associated with the recognition and release of the client protein for metalation [44,45]. Both domains are connected by a linker that in COG0523 proteins grouped in cluster 1 has the consensus sequence PETEEYG [44]. These linker residues are also present in the ZigA protein of *B. ovis* (Figure 2).

The nucleotide sequence of the *zigA-zur* cluster (Figure 1) was determined for parental *B. ovis* PA. It only shows 8 nt substitutions (99.8% identity) when compared to the corresponding locus of *B. abortus* 2308 (Supplementary Figure S1A). None of these differences generate premature translation stop codons in protein encoding regions and only one single-nucleotide difference between both species is observed in the intergenic *znuA-znuC* region (Supplementary Figure S1A).

Considering that the *B. ovis* ZigA displays significant identity with proteins involved in Zn homeostasis in other bacteria that contribute to histidine catabolism [26], orthologs of *hisB* (involved in the histidine biosynthesis pathway; *BOV_2001*) [46], *hutH* (part of the histidine utilization operon; *BOV_A0872*) and *hutC* (described as a transcriptional repressor of the *hut* operon in *B. abortus* [47]; *BOV_A0869*) were identified in the *B. ovis* genome and included in the transcriptomic analysis performed in this work.

The *yciC* gene of *A. tumefaciens*, the ortholog of the *B. ovis zigA* gene, is transcribed in the opposite orientation to the *troCBA* operon (*Atu3178*, *Atu3179*, *Atu3180*) that encodes an ABC-type zinc uptake system that is independent of the classical *znuABC* system [43]. Accordingly, the presence of *troCBA* orthologs in the *B. ovis* genome was investigated, but the closest genes in *B. ovis* were those of the *znuACB* operon and no additional related genes were detected. No orthologs for the *A. tumefaciens* ZinT zinc-binding periplasmic protein (*Atu1049*), which is also regulated by Zur [17], were detected in the *B. ovis* genome.

The *znuD* gene is part of the Zur regulon in *Neisseria meningitidis* (*nmb0964* gene), *Pseudomonas aeruginosa* (*PA0781* gene) and *A. baumannii* (*A1S_2892*) and is highly transcribed under zinc-limiting conditions [29,30,48,49]. ZnuD is a TonB-dependent outer membrane (OM) receptor that shows some sequence homology with *B. ovis* BOV_1306, which is also annotated in the genome as a putative TonB-dependent OM receptor. Accordingly, expression studies performed in this work included *BOV_1306* together with genes for two other putative TonB-dependent OM receptors (*BOV_A0941* and *BOV_A1093*) detected in the *B. ovis* genome and *BOV_1612*, which is the first gene of the *exbB-exbD-tonB* operon. Since TonB-mediated transport is associated with the uptake of bacterial siderophores and the siderophore yersiniabactin also functions as a zinc transporter [50,51], the *BOV_A0013* gene of *B. ovis* PA was also included in the transcriptomic assays. This gene is the ortholog of the *dhbC* gene of *B. abortus* 2308, which is involved in the biosynthesis of the monocatechol siderophore 2,3-dihydroxybenzoic acid (DHBA) [52] and brucebactin, a second *Brucella* siderophore derived from DHBA and structurally more complex [53].

### 3.2. Growth of the B. ovis Strains with Mutations in Genes Related to Zinc Import Is Not Impaired When They Are Cultured in the Presence of the Zn^2+^ Chelator TPEN

**Figure 3:**
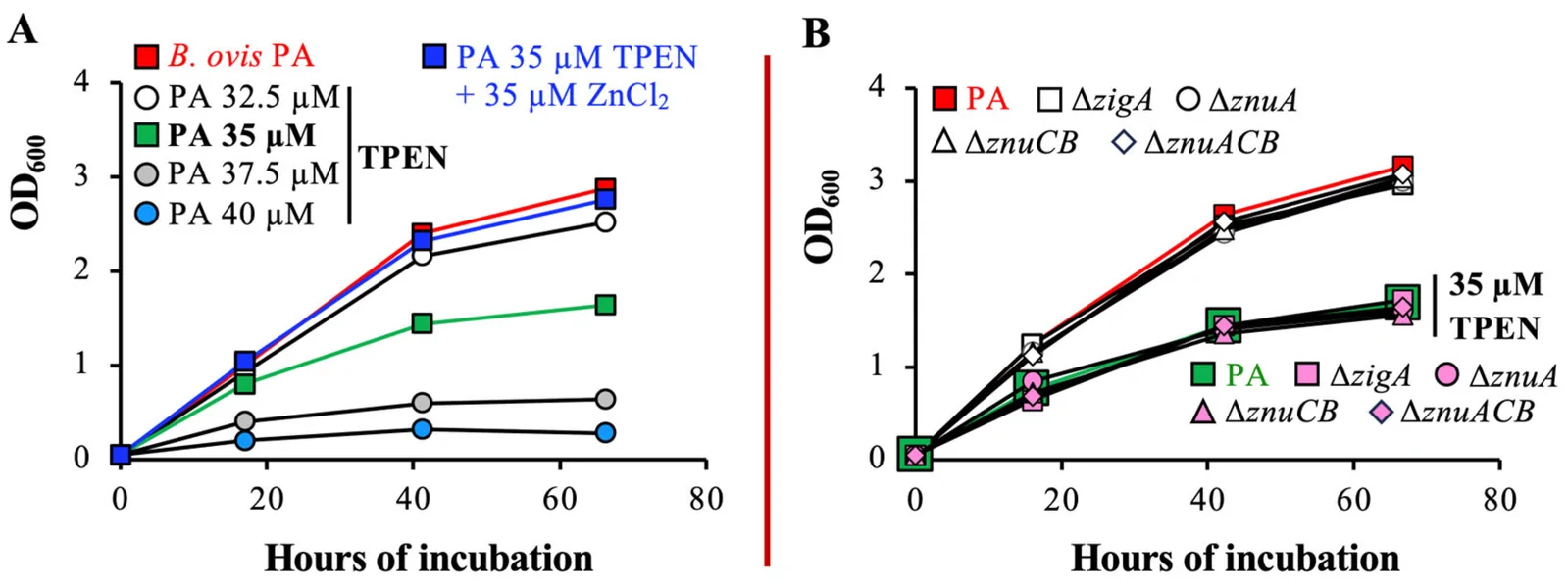
Growth curves of parental *B. ovis* PA in the presence of several concentrations of the zinc chelator TPEN (**A**) and of *B. ovis* PA mutant strains in the presence of 35 µM TPEN (**B**). In panel (**A**), incubation with 35 µM ZnCl_2_ restores growth of *B. ovis* PA cultured with 35 µM TPEN (blue).

Because of its fastidious nature and unknown nutritional requirements, a defined minimal medium is not available for culturing *B. ovis* [54]. Therefore, to mimic zinc-restricted conditions, the Zn^2+^ chelator TPEN [20,55,56] was added to the culture medium. To determine the optimal TPEN concentration for subsequent studies, growth curves of parental *B. ovis* PA were performed in TSB-YE-HS supplemented with different TPEN concentrations (Figure 3A).

Based on the experimental results shown in Figure 3A, 35 µM TPEN was chosen as the preferred concentration to analyze the mutant strains, as it showed some degree of growth inhibition of the parental strain that was abolished by adding 35 µM ZnCl_2_ to the culture medium. The range of TPEN tolerance susceptibility of *B. ovis* PA is tight since, while in the presence of 32.5 µM TPEN its growth is almost unaffected, a concentration of 37.5 µM TPEN clearly impairs its growth (Figure 3A). Surprisingly, growth curves of all mutant strains analyzed in this work were equivalent to those of the parental strain when cultured in the absence or in the presence of TPEN (Figure 3B and Section 3.5). These results suggest the existence of additional mechanisms, other than the ZnuABC system, allowing zinc acquisition under these experimental conditions.

### 3.3. Growth in the Presence of TPEN Alters Expression of Genes Related to Zinc Homeostasis and Histidine Metabolism in B. ovis PA and Reveals Cotranscription of znuA and zigA

**Figure 4:**
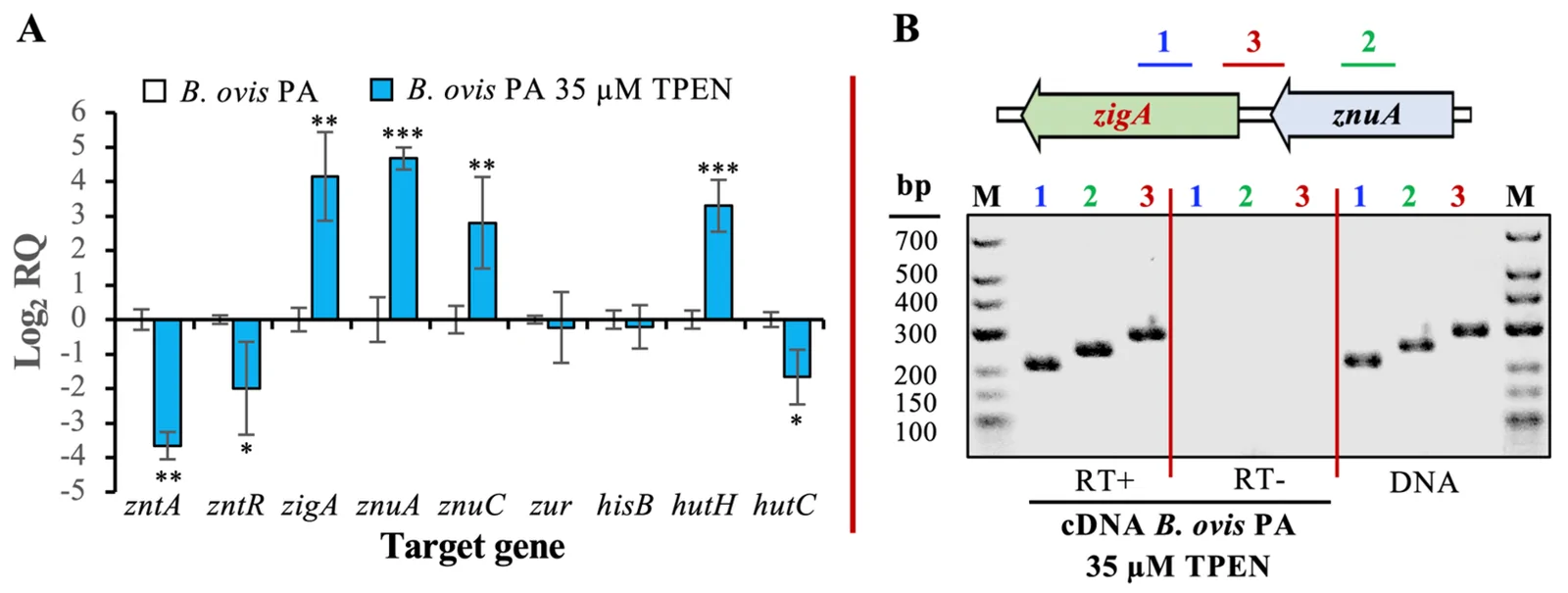
Expression of genes related to zinc and histidine homeostasis in parental *B. ovis* PA cultured for 16 h in the presence of 35 µM TPEN (**A**), and end-point RT-PCR reactions targeting *zigA*, *znuA* or their intergenic region in cDNA obtained from *B. ovis* PA cultured in the presence of 35 µM TPEN for 16 h (**B**). In panel (**A**), statistically significant differences (*p* ≤ 0.05) when compared to *B. ovis* PA cultured in normal medium are marked with asterisks, * *p* ≤ 0.05; ** *p* ≤ 0.01; *** *p* ≤ 0.001. In panel (**B**), regions amplified by end-point RT-PCR (from cDNA resulting from reactions with or without RT, or from DNA) in each lane of the electrophoresis gel are marked with the respective colored lines in the schematic of the upper part of the panel; primer pairs used were zigAF2-EP + zigART-R (1), znuART-F + znuAR2-EP (2) and zigAF-EP + znuAOVL-R (3); lanes M correspond to the O’GeneRuler Low Range DNA ladder (Thermo Scientific, Vilnius, Lithuania).

As expected, exposure of *B. ovis* PA to TPEN resulted in increased transcription of zinc import genes (*znuA* and *znuC*) and decreased transcription of zinc export genes (*zntA* and *zntR*). But notably, this exposure had no impact on the gene encoding the zinc-responsive regulator Zur and highly increased transcription of *zigA* (Figure 4A). However, these observations contrast with the absence of growth defects mentioned above for the mutant strains studied in this work in the presence of TPEN (Figure 3B and Section 3.5).

TPEN exposure did not modify the expression of *hisB* (gene of the biosynthetic pathway of histidine), but elevated transcription of the histidine utilization gene *hutH* and reduced transcription of the gene encoding HutC that is a repressor of *hut* genes (Figure 4A). These experimental findings align with the proposed link between zinc homeostasis and histidine metabolism that has been reported for other bacteria [26] and advance a new area for future research exploring whether this connection also exists in the genus *Brucella*.

Since *zigA* has not been studied previously in the genus *Brucella* and to determine whether it constitutes a monocistronic operon or is cotranscribed with *znuA*, end-point RT-PCR reactions were performed with primer pairs targeting intragenic regions of *zigA* (zigAF2-EP *+* zigART-R) and *znuA* (znuART-F + znuAR2-EP) or their intergenic region (zigAF-EP + znuAOVL-R) (Supplementary Table S1). The results of these analyses indicate that *znuA* and *zigA* constitute a polycistronic operon and display maximum transcription in response to zinc deprivation (Figure 4B, Supplementary Figure S1B).

### 3.4. B. ovis Zur Is a Transcriptional Repressor of zigA, znuA and the znuCBzur Operon, and Its Absence in the Δzur Mutant Significantly Increases Levels of the ZnuA Protein

**Figure 5:**
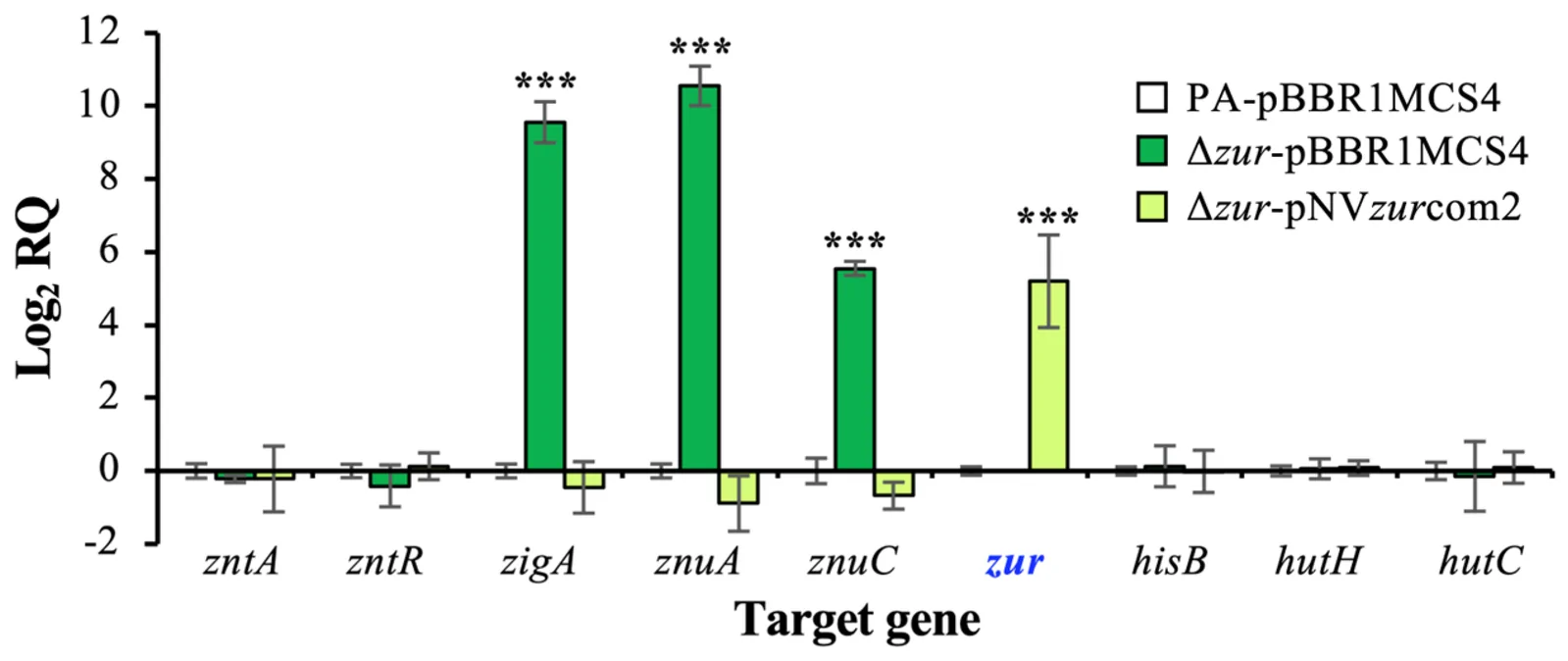
Expression of genes related to zinc and histidine homeostasis in *B. ovis* PA-pBBR1MCS4, the Δ*zur*-pBBR1MCS4 mutant and the same mutant complemented with wild-type *zur*. Expression of *zur* in the Δ*zur* mutant is not represented (blue) and statistically significant differences (*p* ≤ 0.05) when compared to *B. ovis* PA-pBBR1MCS4 are marked with asterisks, *** *p* ≤ 0.001.

Transcription levels in normal medium of genes related to zinc and histidine homeostasis were evaluated by RT-qPCR in the Δ*zur*-pBBR1MCS4 mutant in comparison to those of *B. ovis* PA-pBBR1MCS4 and the complemented mutant Δ*zur*-pNV*zur*com2. The mutant strain showed highly increased levels of *zigA*, *znuA*, and *znuC*, while the other genes exhibited expression levels like those of *B. ovis* PA-pBBR1MCS4. The complemented mutant Δ*zur*-pNV*zur*com2 behaved as *B. ovis* PA-pBBR1MCS4 except for *zur*, whose expression level was significantly higher in the complemented strain in accordance with the multicopy nature of pBBR1MCS4 (Figure 5). Overexpression of *zigA* and *znuA* in the Δ*zur* mutant, when compared to the parental strain, was also evident in the end-point RT-PCR assays, as both genes were detected in the Δ*zur* mutant while their detection in the parental strain required the presence of TPEN in the culture medium (Figure 4B, Supplementary Figure S1B).

**Figure 6:**
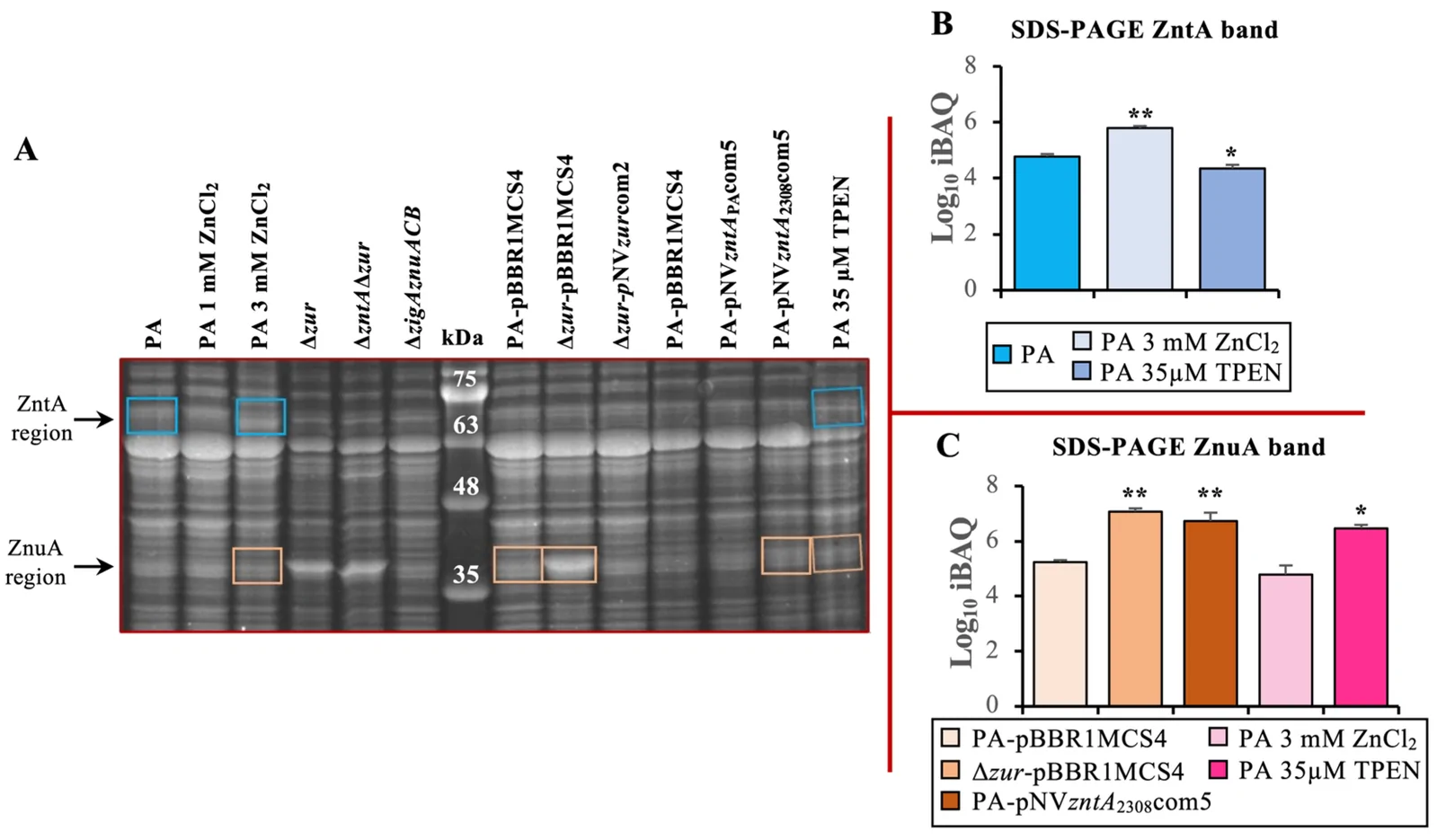
Protein profile after SDS-PAGE of *B. ovis* PA and its derived mutants (**A**), and abundance of proteins ZntA (**B**) or ZnuA (**C**) determined, after reversed-phase LC–MS/MS, in selected strains and culture conditions. In panel (**A**), pre-stained protein marker VI (Applichem-Panreac, Barcelona, Spain) is shown and regions analyzed by reversed-phase LC–MS/MS are framed in blue (ZntA region) or orange (ZnuA region). In panels (**B**,**C**), statistically significant differences (*p* ≤ 0.05) when compared to *B. ovis* PA (**B**) or *B. ovis* PA-pBBR1MCS4 (**C**) are marked with asterisks, * *p* ≤ 0.05; ** *p* ≤ 0.01.

Selected mutant and complemented strains, *B. ovis* PA overexpressing the Zn^2+^ exporter ZntA (from *B. ovis* or *B. abortus*), and *B. ovis* PA cultured in the presence of 35 µM TPEN or 1 or 3 mM ZnCl_2_ were submitted to SDS-PAGE to evaluate their protein profile (Figure 6A, Supplementary Figure S1C).

Although incubation of parental *B. ovis* PA in the presence of 1 mM ZnCl_2_ did not provide a differential protein pattern in SDS-PAGE, which is in agreement with previous results [21], when the concentration of ZnCl_2_ was increased to 3 mM, a protein band of approximately 70 kDa became apparent when compared to the results obtained in normal culture conditions (Figure 6A, Supplementary Figure S1C). The protein was identified by reversed-phase LC-MS/MS as the Zn^2+^ exporter ZntA and showed iBAQ values significantly higher than those observed in normal culture conditions (Figure 6B). On the contrary, iBAQ values of ZntA in *B. ovis* PA cultured with 35 µM TPEN were significantly lower than those measured in control culture conditions (Figure 6B).

Additionally, a markedly overrepresented protein of about 35 kDa was detected in the Δ*zur* mutant and the Δ*zntA*Δ*zur* double mutant, but not in the complemented Δ*zur* mutant (Figure 6A). By reversed-phase LC-MS/MS, this protein was identified as ZnuA in the Δ*zur*-pBBR1MCS4 mutant and showed iBAQ values significantly higher than those detected in *B. ovis* PA-pBBR1MCS4 (Figure 6C). ZnuA was also significantly overrepresented in *B.* ovis PA cultured with 35 µM TPEN and, aligning with previous results [21], in *B. ovis* PA-pN*zntA*_2308_com5 that overexpresses the ZntA Zn^2+^ exporter (Figure 6C).

Despite the highly increased transcription of *zigA* in the Δ*zur* mutant (Figure 5), no protein was clearly overrepresented in this mutant in the region of 45 kDa predicted for ZigA (Figure 6A). This difference with ZnuA could be related to different protein stabilities, since the ProtParam tool (Expasy, Swiss Institute of Bioinformatics) classified ZigA as an unstable protein, contrarily to what was predicted for ZnuA.

### 3.5. B. ovis PA and the ΔzigAznuACB Mutant Cultured with TPEN Show Similar Zinc Content, While the ΔzntA and ΔzntAΔzur Mutants Contain Higher Zn Levels than the Parental Strain When Cultured with ZnCl_2_

**Figure 7:**
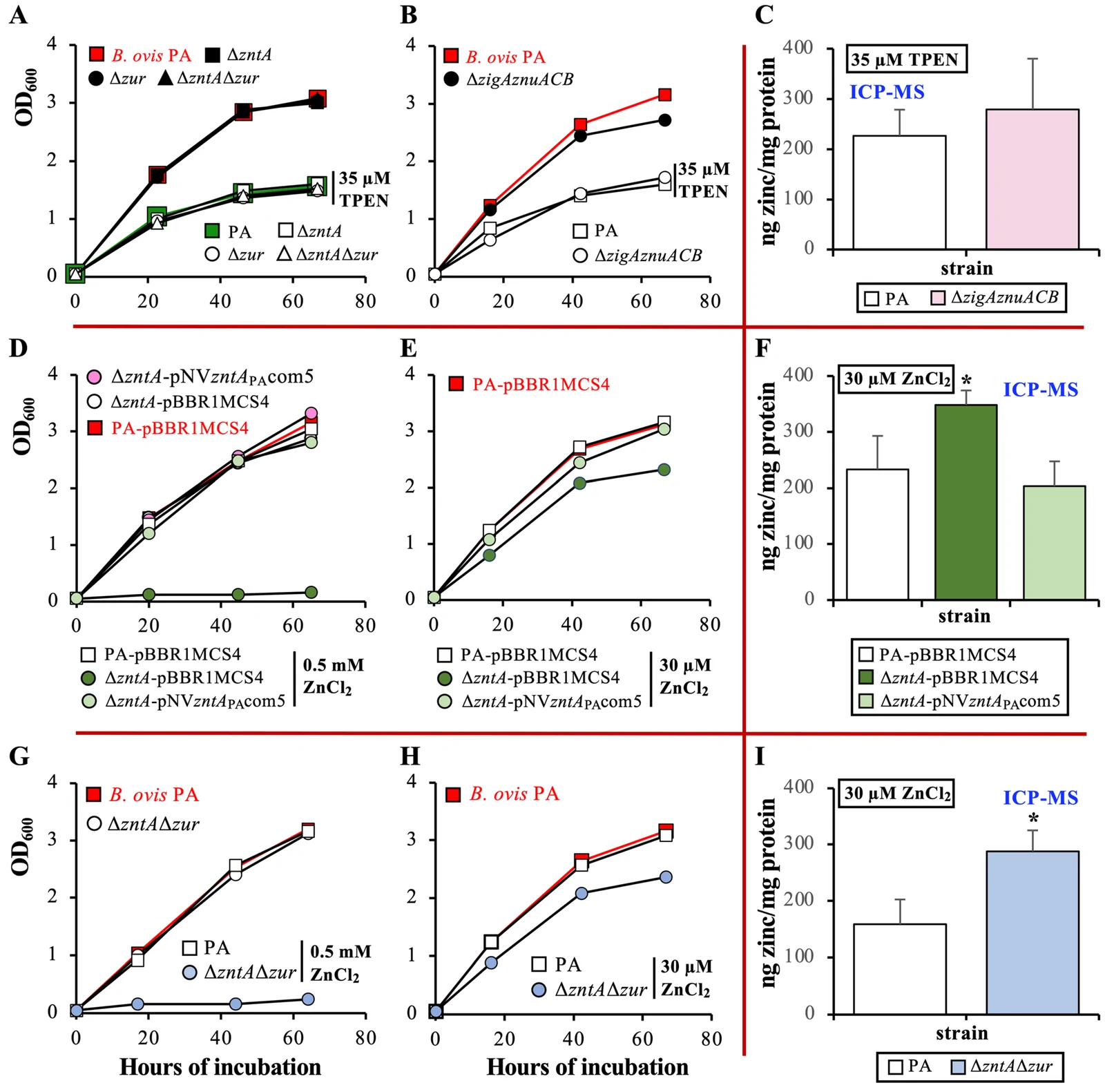
Growth curves of *B. ovis* PA and the Δ*zntA*, Δ*zur*, Δ*zntA*Δ*zur* (**A**) and Δ*zigAznuACB* (**B**) mutants with 35 µM TPEN, and evaluation by ICP-MS of Zn levels in parental *B. ovis* PA and the Δ*zigAznuACB* mutant (**C**). Growth curves of *B. ovis* PA-pBBR1MCS4, the Δ*zntA*-pBBR1MCS4 mutant and the Δ*zntA* mutant complemented with wild-type *zntA* (**D**,**E**) or *B. ovis* PA and the Δ*zntA*Δ*zur* double mutant (**G**,**H**) in the presence of 0.5 mM (**D**,**G**) or 30 µM ZnCl_2_ (**E**,**H**), and evaluation by ICP-MS of Zn levels in these strains cultured for 16 h in the presence of 30 µM ZnCl_2_ (**F**,**I**). In panels (**C**,**F**,**I**), statistically significant differences (*p* ≤ 0.05) when compared to *B. ovis* PA (**C**,**I**) or *B. ovis* PA-pBBR1MCS4 (**F**) are marked with asterisks, * *p* ≤ 0.05.

Growth in the presence of 35 µM TPEN was also analyzed for the Δ*zntA* mutant of *B. ovis* PA, which is defective in a powerful zinc exporter [21], for the Δ*zur* mutant, which lacks the Zur repressor and consequently overexpresses genes of the *zigAznuACB* locus (Figure 5), and for the double Δ*zntA*Δ*zur* mutant. These three mutants showed the same behavior as the parental strain (Figure 7A), as was also the case for the Δ*zigAznuACB* mutant (Figure 7B), which lacks the entire ZnuABC-predicted zinc uptake system together with the newly identified *zigA* gene (Figure 1). Since this mutant behaved like the parental strain in TPEN-containing culture medium, zinc levels in the parental strain and the Δ*zigAznuACB* mutant were assayed by ICP-MS, but no differences were detected between these strains under the conditions of the assay (Figure 7C).

Previous results demonstrated that the Δ*zntA* mutant of *B. ovis* PA tolerated ZnCl_2_ concentrations about 100 times lower than the parental strain, a defect that was abolished by complementation with wild-type *zntA* [21]. This phenotype was confirmed in this work using 0.5 mM ZnCl_2_, a concentration that prevented growth of the Δ*zntA* mutant but not that of the parental strain or the complemented mutant (Figure 7D). Considering these results, zinc levels were also evaluated by ICP-MS in *B. ovis* PA-pBBR1MCS4, the Δ*zntA*-pBBR1MCS4 mutant and the Δ*zntA*-pNV*zntA*_PA_com5 strain (mutant strain complemented with wild-type *zntA* of *B. ovis* PA) that were cultured for 16 h in the presence of 30 µM ZnCl_2_, a concentration that did not affect the growth of the parental and complemented strains and that slightly affected the growth of the Δ*zntA* mutant (Figure 7E). The Δ*zntA*-pBBR1MCS4 displayed significantly higher levels of zinc than the parental strain and the Δ*zntA* mutant complemented with the wild-type gene (Figure 7F). Similar results to those obtained with the single Δ*zntA* mutant were obtained with the Δ*zntA*Δ*zur* double mutant (Figure 7G–I). Complementation of this double mutant with wild-type *zntA*, but not with wild-type *zur*, restored the growth pattern of parental *B. ovis* PA (Supplementary Figure S2A,B). The mutants of the *zigA-znuACBzur* locus obtained in this work behaved as the parental strain in the presence of 0.5 mM ZnCl_2_ (Supplementary Figure S2C,D).

### 3.6. The Siderophore Biosynthesis Gene BOV_A0013 Exhibits Significantly Reduced Transcription in Response to TPEN or Excess Cadmium, but Not in Response to Excess Zinc

Siderophores and their energized import system, frequently constituted by OM receptors linked to a cytoplasmic membrane complex containing the TonB-ExbB-ExbD proteins [57], are intended to facilitate iron import. But some of them have also been linked to zinc acquisition in other Gram-negative bacteria [51,58]. Therefore, *B. ovis* PA representative genes of the siderophore biosynthetic pathway (*BOV_A0013*) and genes predicted to be related to TonB systems (*BOV_1306*, *BOV_1612*, *BOV_A0941* and *BOV_A1093*) were included in the transcriptomic analysis by RT-qPCR.

**Figure 8:**
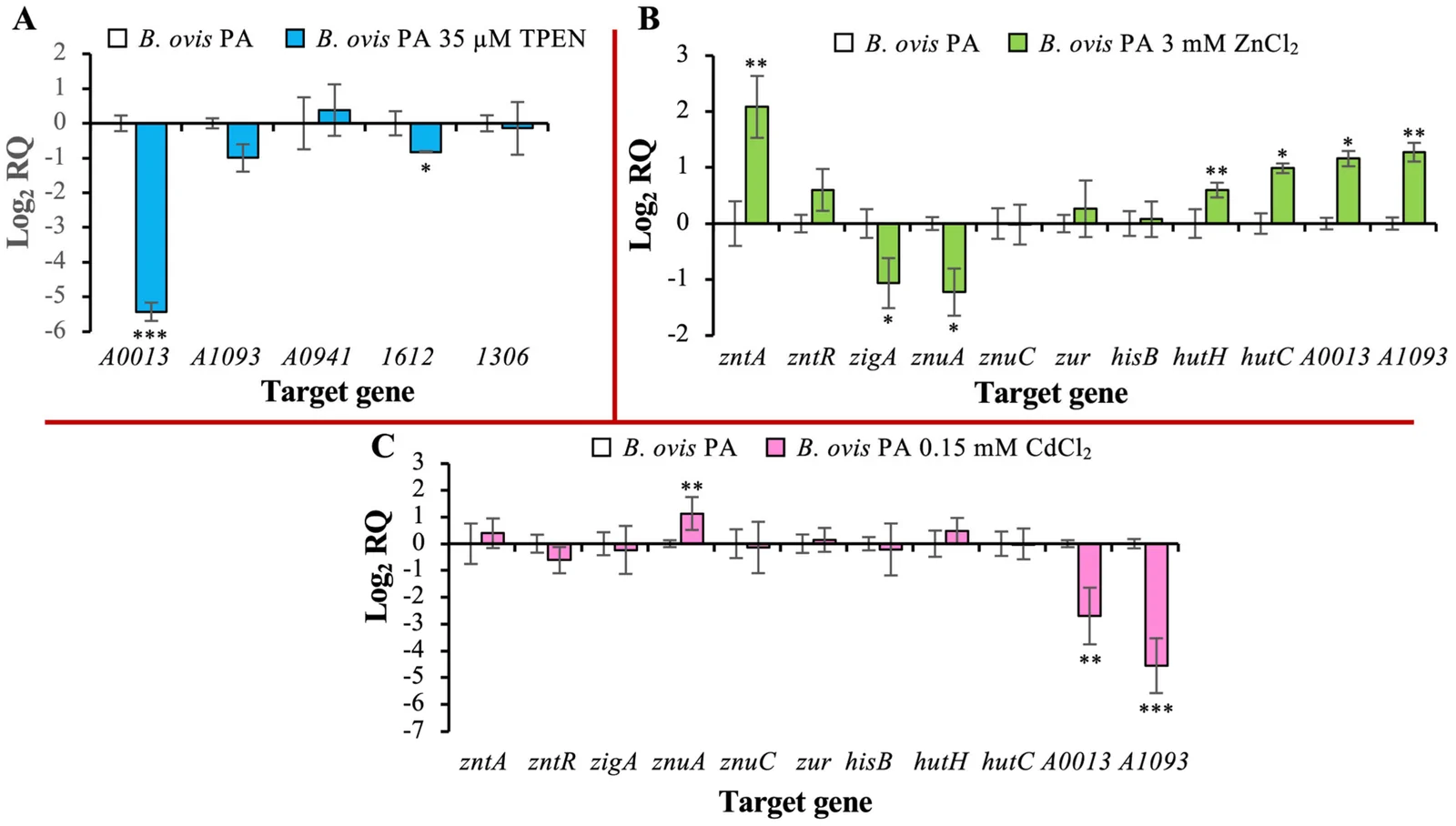
Expression of genes related to zinc and histidine homeostasis, siderophore biosynthesis and/or TonB-mediated transport in *B. ovis* PA cultured in the presence of 35 µM TPEN (**A**), 3 mM ZnCl_2_ (**B**) or 0.15 mM CdCl_2_ (**C**). Statistically significant differences (*p* ≤ 0.05) when compared to *B. ovis* PA cultured in normal culture medium are marked with asterisks, * *p* ≤ 0.05; ** *p* ≤ 0.01; *** *p* ≤ 0.001.

Surprisingly, a strong reduction in the transcription level of the siderophore biosynthetic gene *BOV_A0013* (mean log_2_ RQ values of −5,42, *p* ≤ 0.001) was detected in *B. ovis* PA cultured with TPEN (Figure 8A). The other four genes did not display important differences between both culture conditions, although a slight reduction (mean log_2_ RQ values of −0.82, *p* ≤ 0.05) was observed for *BOV_1612* (Figure 8A).

Transcription of genes related to zinc and histidine homeostasis, as well as *BOV_A0013* and *BOV_A1093*, was also evaluated in the parental strain incubated in the presence of 3 mM ZnCl_2_ or 0.15 mM CdCl_2_. This latter compound was included in the study because the ZntA zinc exporter of *B. ovis* PA is also a strong cadmium exporter [21].

As expected according to previous results [21], *zntA* showed increased transcription in *B. ovis* PA cultured in the presence of 3 mM ZnCl_2_ (Figure 8B). Moreover, the transcription levels of *zigA* and *znuA* were reduced (*p* ≤ 0.05) in the same culture conditions (Figure 8B). This fact contrasts with previous results where transcription of *znuA* in *B. ovis* PA cultured in the presence of ZnCl_2_ was not distinguishable from that detected in normal culture medium [21]. However, it must be noted that these previous results [21] were recorded with a ZnCl_2_ concentration three times lower than that used in the present work (Figure 8B). Increased transcription of *hutH*, *hutC* and *BOV_A1093* was also detected in *B. ovis* PA cultured in the presence of 3 mM ZnCl_2_ (Figure 8B).

Contrary to the results obtained with ZnCl_2_, culture with 0.15 mM CdCl_2_ slightly increased transcription of *znuA* and reduced the transcription levels of *BOV_A0013* and *BOV_A1093* (Figure 8C).

### 3.7. Culture of B. ovis PA in the Presence of the Metal Chelator EDTA Reveals a Transcription Profile Comparable to That Obtained with TPEN

**Figure 9:**
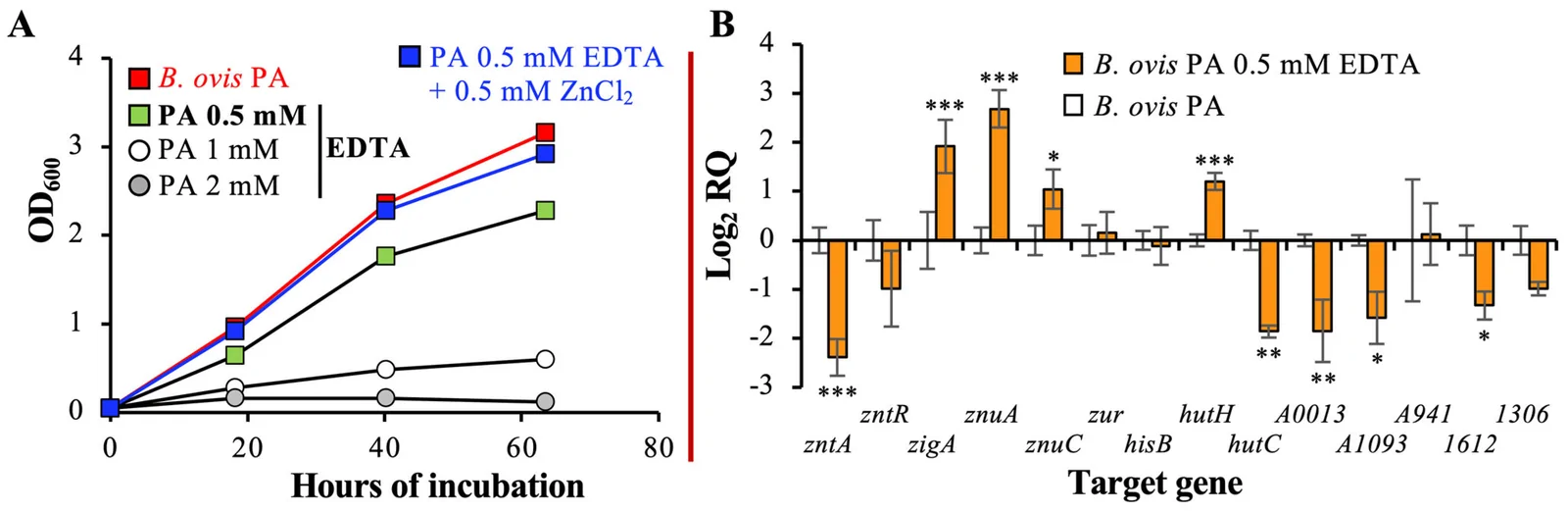
Growth curves of parental *B. ovis* PA in the presence of several concentrations of the metal chelator EDTA (**A**) and expression of genes related to zinc and histidine homeostasis, siderophore biosynthesis and/or TonB-mediated transport in *B. ovis* PA cultured in the presence of 0.5 mM EDTA (**B**). In panel (**A**), incubation with 0.5 mM ZnCl_2_ restores growth of *B. ovis* PA cultured with 0.5 mM EDTA (blue). In panel (**B**), statistically significant differences (*p* ≤ 0.05) when compared to *B. ovis* PA cultured in normal culture medium are marked with asterisks, * *p* ≤ 0.05; ** *p* ≤ 0.01; *** *p* ≤ 0.001.

Since no growth differences were observed in TPEN-containing culture medium among the *B. ovis* PA mutants defective in genes related to the zinc import and export systems, EDTA was also evaluated as an additional metal chelator with *B. ovis* PA and the different mutants obtained in this work. To choose the optimal EDTA concentration to be used, growth curves of parental *B. ovis* PA were made in TSB-YE-HS supplemented with different EDTA concentrations. According to the results (Figure 9A), 0.5 mM EDTA was selected as the concentration to perform susceptibility and gene expression assays with the *B. ovis* parental and mutant strains. The defective growth of *B. ovis* PA in the presence of 0.5 mM EDTA was abolished by the addition of 0.5 mM ZnCl_2_ (Figure 9A).

Parental *B. ovis* PA cultured with 0.5 mM EDTA showed a transcription pattern of genes related to zinc and histidine homeostasis and to siderophore biosynthesis and TonB-related transport (Figure 9B) equivalent to that observed when cultured with 35 µM TPEN (Figure 4A and Figure 8A). Specifically, when compared to normal culture conditions, *zntA* (coding for the zinc exporter) was undertranscribed, while *zigA*, *znuA* and *znuC* (genes of the zinc import locus) were overtranscribed in *B. ovis* PA cultured with 0.5 mM EDTA. Reduced transcription was also observed for *BOV_A0013*, *BOV_A1093* and *BOV_1612* (genes linked to siderophore biosynthesis and TonB-related transport). Genes coding for HutH (histidine utilization pathway) and HutC (repressor of *hut* genes) showed higher and lower transcription levels, respectively, in response to EDTA treatment (Figure 9B).

### 3.8. The ΔznuACB and ΔzigAznuACB Mutants Display Growth Defects in Response to EDTA Treatment That Can Be Rescued by the Addition of ZnCl_2_ and Partially Rescued with NiCl_2_, MnCl_2_, CoCl_2_ or FeCl_2_

**Figure 10:**
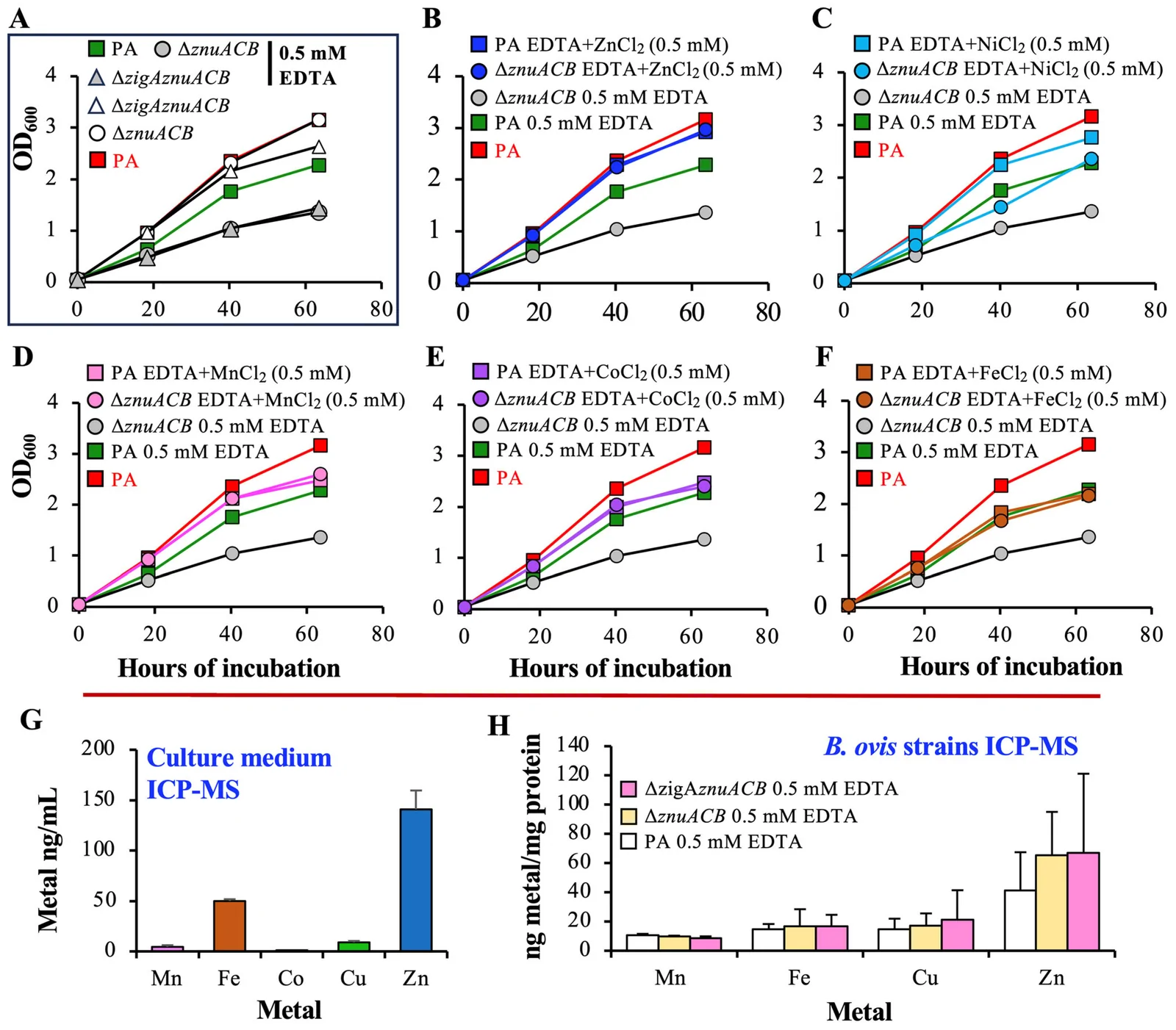
Growth curves of *B. ovis* PA and the Δ*znuACB* or Δ*zigAznuACB* mutants in the presence of 0.5 mM EDTA (**A**) or with 0.5 mM EDTA plus 0.5 mM ZnCl_2_ (**B**), NiCl_2_ (**C**), MnCl_2_ (**D**), CoCl_2_ (**E**), or FeCl_2_ (**F**). ICP-MS metal concentrations in TSB-YE-HS culture medium (**G**) and in *B. ovis* PA and the Δ*znuACB* and Δ*zigAznuACB* mutants after 16 h of culture with 0.5 mM EDTA (**H**). Growth curves with the Δ*zigAznuACB* mutant, equivalent to those obtained with the Δ*znuACB*, are shown in Supplementary Figure S3. Addition of CuCl_2_ or MgCl_2_ did not restore growth of parental *B. ovis* PA or mutant strains cultured in TSB-YE-HS 0.5 mM EDTA (Supplementary Figure S3).

Growth in culture medium supplemented with 0.5 mM EDTA was analyzed for all of the *B. ovis* strains examined in this work. All mutants behaved like the parental strain except for the Δ*znuACB* and Δ*zigAznuACB* mutants, which displayed an impaired growth (Figure 10A, Supplementary Figure S2E). Addition of 0.5 mM ZnCl_2_ recovered the parental phenotype without EDTA, but a partial recovery was also observed when NiCl_2_, MnCl_2_, CoCl_2_ or FeCl_2_ were added to the culture medium, with the latter three reaching the levels of the parental strain cultured in the same conditions (Figure 10B–F, Supplementary Figure S3A–E). Additionally, 0.5 mM CuCl_2_ or MgCl_2_ was tested, but no differences were found when compared to the Δ*znuACB* and Δ*zigAznuACB* mutants cultured with 0.5 mM EDTA alone (Supplementary Figure S3F–I).

In order to determine if the results described in the paragraph above could be related to metal availability in the *B. ovis* strains, the levels of Zn, Mn, Fe, Cu and Co present in parental *B. ovis* PA and the Δ*znuACB* and Δ*zigAznuACB* mutants cultured with 0.5 mM EDTA were determined by ICP-MS and compared to those present in the parental strain in normal culture conditions. Profiles for these five metals were also determined for the TSB-YE-HS culture medium, with Zn being the most abundant metal with levels of 141 ± 19 ng/mL (Figure 10G). The concentration of Fe (50 ± 2 ng/mL) was approximately three times lower than that of Zn in this medium, while Cu, Mn and Co were detected to a lesser extent (Figure 10G). Zn was also the most abundant metal detected in the *B. ovis* strains examined, while Co was under the detection limit (Figure 10H). However, no statistically significant differences could be demonstrated for any of the assayed metals (*n* = 3) in these strains when they were cultured in the presence of EDTA (Figure 10H).

### 3.9. The ΔznuACB and ΔzigAznuACB Mutants Exhibit Reduced Expression of the Genes Evaluated in This Work Under EDTA Treatment

**Figure 11:**
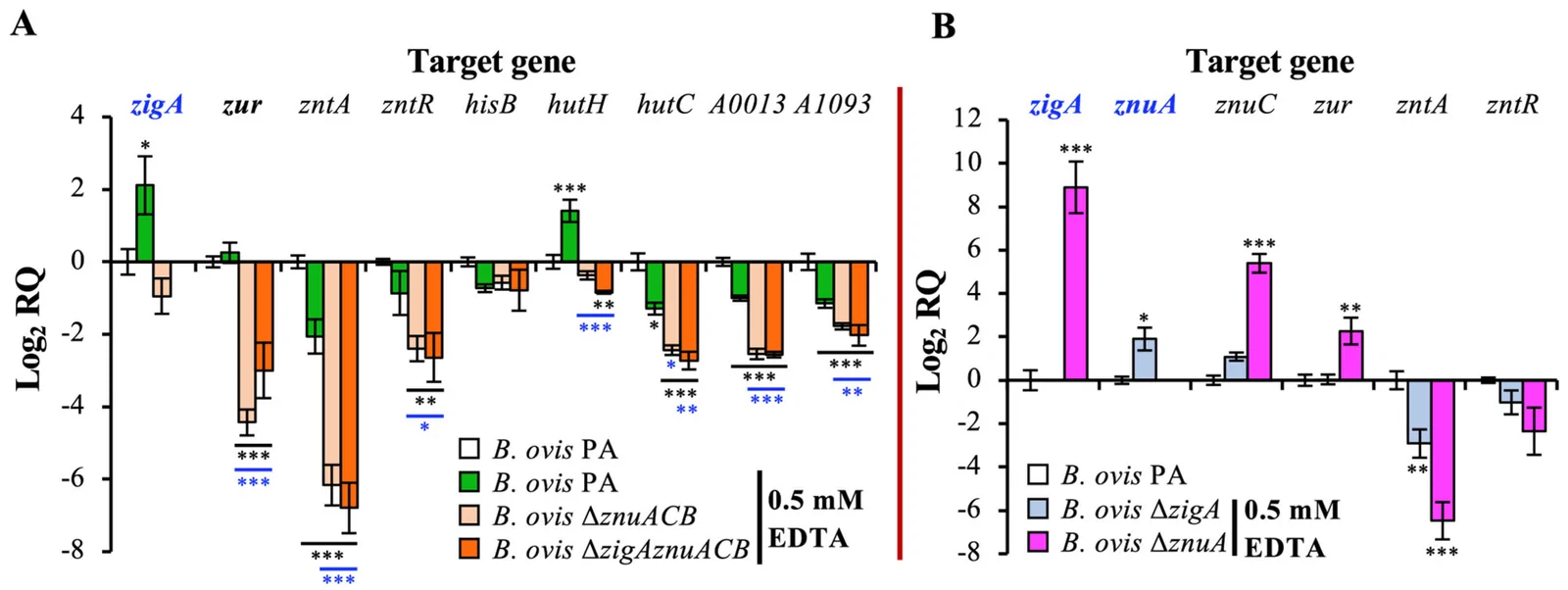
Expression of genes related to zinc and histidine homeostasis, siderophore biosynthesis and TonB-mediated transport in *B. ovis* PA and the Δ*znuACB* and Δ*zigAznuACB* mutants cultured in the presence of 0.5 mM EDTA (**A**), and expression of genes related to zinc homeostasis in *B. ovis* PA and the Δ*zigA* and Δ*znuA* mutants (**B**). Target genes in blue correspond to genes that were not evaluated in the corresponding deletion mutant. Statistically significant differences (*p* ≤ 0.05) when compared to *B. ovis* PA cultured in normal culture medium are marked with black asterisks. Statistically significant differences in mutant strains cultured with 0.5 mM EDTA in comparison to *B. ovis* PA cultured with 0.5 mM EDTA are marked with blue asterisks, * *p* ≤ 0.05; ** *p* ≤ 0.01; *** *p* ≤ 0.001.

Considering the growth defects observed for the Δ*znuACB* and Δ*zigAznuACB* mutants in the presence of EDTA, the expression levels of genes related to zinc and histidine homeostasis and to siderophore biosynthesis and TonB-related transport were evaluated in both mutants and in Δ*zigA*, Δ*znuA* and Δ*zigAznuA* mutants cultured with EDTA (Figure 11A,B, Supplementary Figure S4).

In the Δ*znuACB* and Δ*zigAznuACB* mutants cultured in the presence of EDTA, a strong reduction in transcripts for the ZntA zinc exporter was observed (Figure 11A), which suggests that the intracellular zinc levels are substantially decreased in these strains. Zur transcripts are also significantly decreased in both strains, probably due to the absence of the natural promoter region located in the *znuA-znuC* intergenic region (Figure 1). The same reason probably underlies the *zigA* subexpression detected in the Δ*znuACB* mutant (Figure 11A). A significant diminished transcription of *hutH*, *hutC*, *BOV_A0013* and *BOV_A1093* was also detected in both mutants when compared to the parental strain (Figure 11A).

Gene expression was also studied with the Δ*zigA*, Δ*znuA* and Δ*zigAznuA* mutants cultured in the presence of 0.5 mM EDTA. While deletion of *zigA* did not affect transcription of the remaining genes of the locus (Figure 11B), deletion of *znuA* led to a strong increased transcription of *zigA*, *znuC* and *zur* (*znuB*, located between *znuC* and *zur*, was not analyzed) and decreased *zntA* transcription (Figure 11B). Genes linked to histidine metabolism and siderophore-mediated metal transport displayed a similar transcription profile to that observed with *B. ovis* PA cultured with EDTA (Figure 11A, Supplementary Figure S4A). A similar expression pattern was also observed with the Δ*zigAznuA* mutant, except for the genes deleted in this strain (Supplementary Figure S4B).

### 3.10. The ZnuACB Import System Is Not Required for Virulence of B. ovis PA in Mice

**Figure 12:**
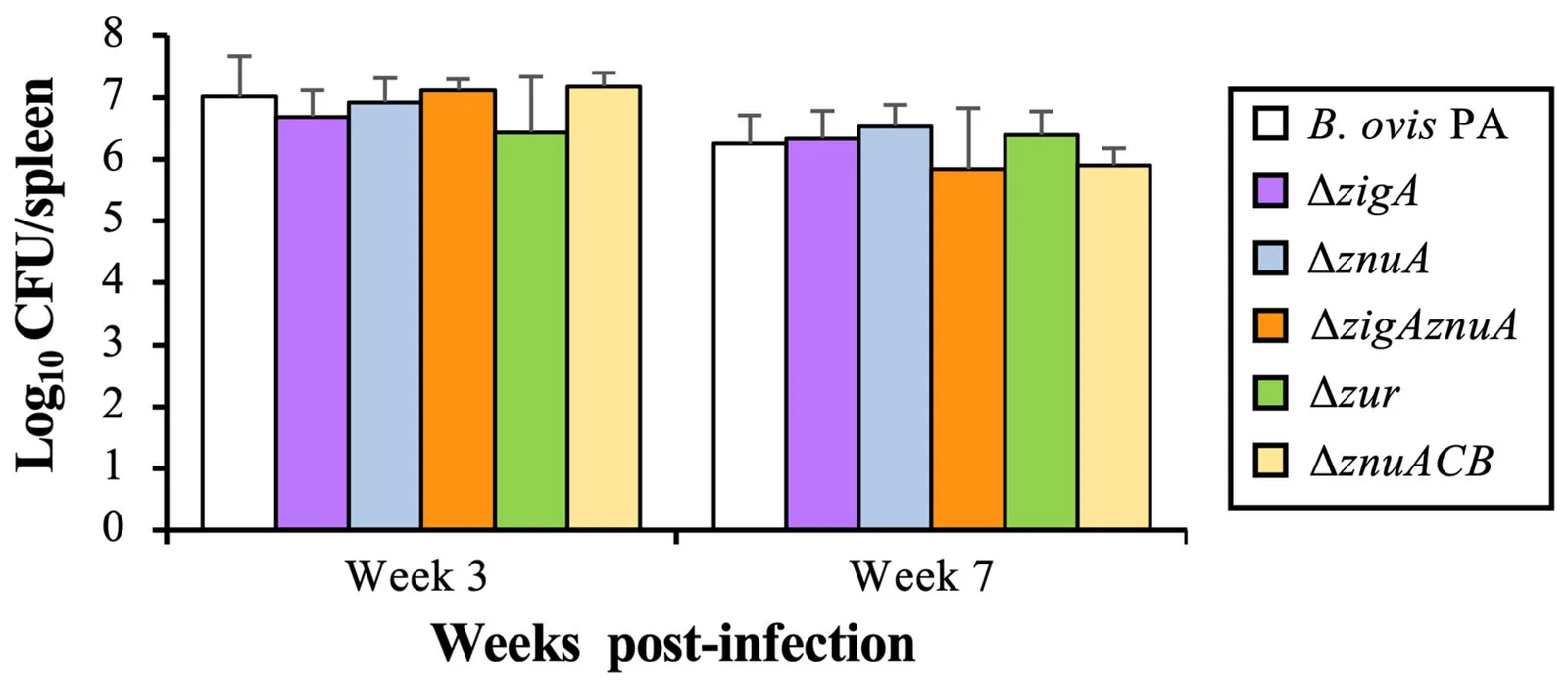
Virulence of the *B. ovis* mutants in mice inoculated intraperitoneally with 10^6^ CFU/mouse. Spleen colonization was evaluated at weeks 3 and 7 post-infection in five mice per strain and time point. Results are represented as means ± SD (*n* = 5) of the log_10_ CFUs/spleen. Statistically significant differences (*p* ≤ 0.05) when compared to *B. ovis* PA were not detected and are not marked.

Based on the results described in previous sections, several single and multiple mutants lacking the zinc-responsive regulator Zur or the zinc import-related proteins ZigA, ZnuA, ZnuC and ZnuB (Figure 1) were evaluated for virulence in the BALB/c mouse model of chronic infection (Figure 12).

Deletion of *znuA* did not affect the virulence of *B. ovis* PA (Figure 12), which contrasts with the results previously described for the *B. abortus* and *B. melitensis* Δ*znuA* mutants that showed a marked attenuation in cellular models and mice [23,24,25]. In fact, the Δ*zigA*, Δ*zigAznuA*, Δ*zur* and Δ*znuACB* mutants also displayed a virulence pattern that was undistinguishable from that observed with the parental strain.

## 4. Discussion

Zinc is an essential micronutrient that plays a crucial role in the functioning of all living organisms, including vertebrate hosts and bacterial pathogens, and is required for structural, regulatory or catalytic properties of many proteins [12]. On the contrary, the predicted high-affinity interactions of zinc with different metal-binding sites [14] results in mismetallation of non-zinc metalloproteins, causing detrimental effects on the cell, in zinc-rich environments [11,12]. Accordingly, intracellular zinc levels must be tightly balanced to preserve cell viability.

To maintain zinc homeostasis, bacterial pathogens, and particularly intracellular pathogens like *Brucella*, must overcome nutritional immunity that is imposed by the host through zinc availability restriction and/or zinc intoxication mechanisms [12]. Studies targeting zinc homeostasis in the genus *Brucella* are few, despite the relevance shown in other bacteria. The ZntA-ZntR system, made up of the zinc exporter ZntA and its transcriptional regulator ZntR, has only been studied in smooth *B. abortus* 2308 [20] and more recently in rough *B. ovis* PA [21]. Moreover, data regarding zinc uptake mediated by the ZnuABC transporter, a main zinc importer in many bacteria, has only been reported in the two smooth species *B. melitensis* and *B. abortus* where the respective Δ*znuA* mutants were found to be attenuated in mice [23,24,25]. Despite the high degree of DNA identity existing among the pathogenic species of the genus *Brucella* [59], a Δ*zntR* mutant of *B. abortus* 2308 was attenuated in mice in the chronic phase of infection [20], while a Δ*zntR* mutant of *B. ovis* PA was not [21]. Attenuation of the *B. abortus* Δ*zntR* mutant was experimentally linked with increased expression of the ZntA zinc exporter [20], but deletion of *zntR* in *B. ovis* PA had the opposite effect with a significant reduction in *zntA* transcription [21]. Considering the differences found between smooth *Brucella* strains and rough *B. ovis* PA regarding the ZntA-ZntR zinc export system [20,21], the main aim of the present work was to characterize the role of the ZnuABC zinc import system of *B. ovis* PA in zinc homeostasis and virulence.

The first finding was the identification of a gene, located at the 3′ end of *znuA* (Figure 1) and never studied in the genus *Brucella*, showing the characteristic motifs of COG0523 zinc metallochaperone GTPases whose expression is regulated by Zur (Figure 2), as ZigA of *A. baumannii* [44]. The *B. ovis* PA *zigA* gene was also demonstrated to be regulated by Zur (Figure 5) and to have increased expression in the presence of TPEN and EDTA chelators (Figure 4A and Figure 9B), while it showed a diminished transcription under zinc excess conditions (Figure 8B). These characteristics suggest that the *B. ovis* PA ZigA protein might play a relevant role in maintaining zinc homeostasis in zinc-depleted environments.

Zur and ZntR are zinc-sensitive regulators governing expression of the main bacterial zinc export–import systems under zinc excess and insufficiency conditions. They bind zinc with high affinity and work coordinately enabling the finely tuned switch-on and switch-off of zinc uptake and efflux systems [60,61]. Specifically, under excess conditions, zinc binds both ZntR (consequently activating expression of the ZntA exporter) and Zur, which represses genes involved in zinc acquisition. This balanced regulation, coupled to the reported almost absence of free zinc in the bacterial cytoplasm despite its relatively high content in the cell, raised the idea of the existence of a pool of chelated zinc that could be mobilized and transferred to essential proteins during zinc starvation [60]. Some bacterial metabolites have been associated with a labile zinc pool, among them the amino acid histidine in *A. baumannii* [12,13,26]. In this bacterium, the zinc metallochaperone GTPase ZigA was reported to contribute to metallation of the histidine ammonia lyase HutH that metabolizes histidine into urocanic acid, which is further converted into glutamate via the activity of other enzymes encoded by the *hut* operon. Given that both urocanic acid and glutamate have less affinity for zinc than histidine, the availability of zinc in the cell increases with histidine catabolism [12,13,26]. Both ZigA and HutH were shown to be required for adaptation to zinc starvation in *A. baumannii*, where *zigA* is closely located in the genome to the *hut* operon [26]. Although this genome distribution is not conserved in *B. ovis* PA (Figure 1), increased expression of *hutH* and decreased expression of *hutC*, which encodes a transcriptional repressor of the *hut* operon in *B. abortus* [47], was observed in zinc starvation conditions induced by the TPEN or EDTA chelators (Figure 4A and Figure 9B). Although these findings are exclusively based on expression studies, and additional research is required, they also suggest a possible link in *B. ovis* PA between histidine catabolism and zinc homeostasis under zinc scarcity conditions. This potential connection would not be mediated, at least directly, by Zur since the Δ*zur* mutant of *B. ovis* PA did not show differences in expression of *hutH* or *hutC*, contrary to the highly increased transcription observed in this mutant for *zigA*, *znuA* and *znuC* (Figure 5).

A clear change in the transcription profile of the classical zinc import–export genes, suggestive of restricted intracellular zinc, was observed for parental *B. ovis* PA grown in the presence of TPEN (Figure 4A). However, unexpectedly, none of its derived mutants for the *zigA-zur* locus (Table 1; Figure 1), or the Δ*zntA* or double Δ*zntA*Δ*zur* mutants (Table 1), displayed an increased growth defect in the presence of 35 µM TPEN when compared to the parental strain (Figure 3B and Figure 7A,B). Because a defined minimal medium is not available for culturing *B. ovis*, a zinc-restricted environment was mimicked with the TPEN chelator, which has been widely used with this purpose in many bacteria [26,29,30,48], including *B. abortus* [20]. However, due to its lipophilic nature, TPEN is described as a membrane-permeable chelator in studies performed with eukaryotic cells [62]. This property could be related to the lack of increased detectable defects in the presence of TPEN for the *B. ovis* PA mutant strains obtained and with the similar zinc content detected in the presence of TPEN for the parental strain and the *ΔzigAznuACB* mutant (Figure 7C). If chelated zinc enters the cell, independently of specific import systems, the metal could be sequestered by bacterial proteins in enough quantity to maintain viability. This could also explain the narrow margin of TPEN concentration that separates normal growth from the absence of replication in *B. ovis* PA (Figure 3A), which could be triggered when the TPEN concentration surpasses the threshold in which the cell would be unable to compete with the chelator for metal binding.

Despite the previous comments, the existence of additional mechanisms for active or passive zinc acquisition in *B. ovis* seems probable, particularly considering that the parental strain and all mutant strains showed a similar growth pattern also in the absence of TPEN. This possibility for additional zinc uptake mechanisms also seems plausible in the smooth *Brucella* species analyzed in previous reports, since the Δ*znuA* mutants of *B. abortus* strains 2308 and 544 and *B. melitensis* 16M also grew in rich medium [23,24,25] and the *B. abortus* 544 mutant showed limited growth defects in culture medium chelated with EDTA (the *B. melitensis* 16M Δ*znuA* mutant was not tested under zinc-restricted conditions). The most significant effect of *znuA* deletion was reported with the mutant of *B. abortus* 2308, which could be tested in a low-zinc minimal medium available for this species. In these zinc-restricted conditions, the Δ*znuA* mutant of *B. abortus* 2308 was unable to grow, but supplementation of the minimal medium with zinc restored growth of the mutant strain [25], which also suggests the existence of additional mechanisms for zinc acquisition. However, the Δ*znuA* single mutants of these three smooth *Brucella* strains were attenuated in a mouse model based on splenic counts [23,24,25], which demonstrates the significance of ZnuA for their infectious process and contrasts with the lack of attenuation of the Δ*znuA*, Δ*zigAznuA* and Δ*znuACB* mutants of rough *B. ovis* PA in an equivalent model of infection (Figure 12). These contrasting results do not appear to derive from interspecies differences in the *zigA-zur* locus at the DNA level, as the minor nucleotide substitutions detected only affected one amino acid of each of the five proteins of the locus and one nucleotide in the intergenic *znuA-znuC* region (Supplementary Figure S1A). No additional zinc import systems have been detected in *B. ovis* but a relevant differential characteristic between smooth and rough *Brucella* strains is the absence of O-polysaccharide chains in the bacterial surface. Consequently, membrane permeability to diverse compounds may differ between rough and smooth strains, and other OM components, such as porins, that are more exposed on the surface of *B. ovis* might have a role in zinc uptake. In this respect, differences among *Brucella* species regarding the profile of OM proteins (OMPs) and OM-related properties have been described [38,63,64] and might impact zinc homeostasis mechanisms. Additionally, a genomic island in *B. ovis*, absent from *B. abortus* and *B. melitensis*, encodes an ABC transporter required for virulence in mice and sheep [65,66]. The exact function of this transporter has not been elucidated but it seems to be required for expression of the type-IV secretion system [65], a recognized virulence factor in *Brucella* spp. including *B. ovis* [27]. Although no relationship with zinc uptake has been reported for this ABC transporter, this possibility cannot be discarded, also considering that in the KEGG database, the putative substrate-binding protein associated with this ABC transporter (BOV_0504) is annotated as a peptide/nickel transport system substrate-binding protein. The role in zinc uptake of other ABC transporters of *B. ovis*, and nonfunctional in *B. abortus* and *B. melitensis*, does not seem probable since deletions and/or pseudogenization have considerably reduced their number in *B. ovis* (59 versus 79 ABC systems detected in *B. melitensis*) [67].

Another striking finding obtained during this work was the strong subexpression of the siderophore biosynthesis gene *dhbC* (*BOV_A0013*) in *B. ovis* PA when exposed to TPEN (Figure 8A). This effect, although more moderate, was also replicated in the parental strain after exposure to EDTA and was more pronounced in the mutants for the *znuACB* locus exposed to the same chelator (Figure 9B and Figure 11A, Supplementary Figure S4). These results, which are accompanied by increased transcription of *znuA* and subexpression of the *zntA* zinc exporter gene (Figure 4A and Figure 11, Supplementary Figure S4B), suggest that zinc deprivation leads to a diminished transcription of siderophore biosynthesis genes. Although this effect could be triggered by chelation of other metals by TPEN or EDTA, and the expression of other genes related to iron uptake (that could increase intracellular iron level) has not been evaluated in this work, it must be noted that an opposed effect, with increased transcription of *BOV_A0013*, was observed after exposure to high zinc in the absence of chelators (Figure 8B). Moreover, exposure to cadmium in the absence of chelators also strongly influenced *BOV_A0013* expression but in the opposite direction to zinc (Figure 8C). This gene, as well as *BOV_A1093* that encodes a TonB-related OM receptor, exhibited a strongly diminished transcription in the presence of cadmium that was accompanied by a slight increase in *znuA* transcription suggestive of zinc deprivation conditions in the cell (Figure 8C). Zinc and cadmium interplay was previously described in *B. ovis* since the ZntA zinc exporter was shown to be a powerful cadmium exporter [21] as also reported in other bacterial species [22,68,69]. Accordingly, a link between the regulatory mechanisms controlling the intracellular levels of zinc, iron and cadmium seems to exist in *B. ovis*, as it has been reported in *Salmonella* where accumulation of intracellular cadmium also mimicked zinc starvation conditions [69].

It must also be noted that, in addition to the strong activity as a cadmium exporter discussed above, the contribution of the *B. ovis* ZntA zinc exporter to cobalt, copper and nickel detoxification was also previously evidenced [21]. Moreover, although expression studies with the different mutants of the *zigA-zur* locus suggested increased zinc deprivation when cultured with EDTA (Figure 11, Supplementary Figure S4) and the growth defects observed for the Δ*znuACB* and Δ*zigAznuACB* mutants of *B. ovis* PA in the presence of this chelator were restored by adding ZnCl_2_ to the culture medium, a partial restoration of growth was also observed with other metals (Figure 10, Supplementary Figure S3). The limitations derived from using chelators, with different affinities for several metals, challenge the interpretation of these results, but they also suggest a complex regulatory network governing metal homeostasis in *B. ovis* that merits being evaluated in further studies.

In addition to this initial evidence about the interaction between metal homeostatic mechanisms in *B. ovis*, the identification of a new gene of the Zur regulon in the genus *Brucella* and a potential link between zinc homeostasis and histidine metabolism that also deserves further investigation, a notable observation of this work is the dispensability of the ZnuACB zinc import system for the virulence of *B. ovis*, at least in the mouse model, which contrasts with previous reports of its essential role in smooth *Brucella* species. Some plausible explanations for these in vivo apparent differences, concerning structural differences between *B. ovis* and smooth *Brucella* strains, have already been discussed above. However, the known slower growth of *B. ovis* and its particularities regarding host–pathogen interactions (including a different route of entry into phagocytes and a slower intracellular trafficking until reaching the replicative vacuole) [64,65,70] might also contribute to these apparent differences, constituting a relevant line for future comparative studies. However, considering that virulence has only been evaluated in the mouse model, a different behavior in the natural host cannot be discarded. Further characterization of the *B. ovis* mutants in zinc homeostasis systems in the natural host and/or ovine cell lines would provide a more detailed picture about the role of virulence in these systems.

## 5. Conclusions

Overall, this work provides relevant information about zinc homeostasis in the genus *Brucella*, an area of research largely unexplored in rough *Brucella* species such as *B. ovis*. The identification and characterization of a *zigA* ortholog, shown to be controlled by Zur and upregulated under zinc scarcity conditions, represents the first report in the genus *Brucella* about this gene and its link with zinc homeostasis. Although it is important to consider the limitations of using TPEN and EDTA chelators to induce a zinc-restricted environment, and that the evidence primarily comes from expression studies, the results obtained in this work also suggest a potential connection between histidine catabolism and zinc homeostasis as well as a crosstalk between homeostatic mechanisms of several metals that merit being further explored.

Evidence for additional zinc acquisition mechanisms, other than the ZnuABC system, has been obtained in *B. ovis* that also seem to exist in the smooth *Brucella* species previously analyzed. The full virulence in the mouse model of the *B. ovis* mutant defective in ZnuA, which contrasts with the attenuation previously described in the same model for smooth *Brucella* strains, points to a new differential characteristic between smooth zoonotic *Brucella* species and rough non-zoonotic *B. ovis*, species that are highly homologous at the DNA level, but display relevant differences in pathogenicity and host preference. Although the exact basis for these contrasting results in virulence remains to be elucidated, two plausible explanations may rely on (i) the absence of O-polysaccharide chains in *B. ovis* and other specific characteristic of its OM, which might lead to increased permeability to zinc, and/or (ii) the particular host–pathogen interactions reported for *B. ovis*. However, the presence of additional species-specific zinc uptake mechanisms in *B. ovis* cannot be ruled out and merits further investigation. This work pioneers the characterization of zinc uptake systems in *B. ovis*, opening new lines of research for future studies. The evaluation of the natural host or/and in ovine cell lines of the *B. ovis* mutants defective in zinc uptake and export systems would provide a more accurate view about the role of zinc homeostasis in the virulence of *B. ovis*.

## Figures and Tables

**Table 1:** *B. ovis* bacterial strains used in this work.

Strain Name	Relevant Characteristics	Origin
*B. ovis* PA	Parental strain	BCCN ^1^
*B. ovis* PA-pBBR1MCS4	Parental strain bearing pBBR1MCS4	[21]
*B. ovis* PA-pNVzntA_PA_com5	Parental strain bearing pNV*zntA*_PA_com5	[21]
*B. ovis* PA-pNV*zntA_2308_*com5	Parental strain bearing pNV*zntA*_2308_com5	[21]
*B. ovis* ∆*zntA*	∆*zntA* deletion mutant of *B. ovis* PA	[21]
*B. ovis* ∆*zntA*-pBBR1MCS4	∆*zntA* mutant bearing pBBR1MCS4	[21]
*B. ovis* ∆*zntA*-pNV*zntA*_PA_com5	∆*zntA* mutant complemented with pNV*zntA*_PA_com5 ^2^	[21]
*B. ovis* ∆*zntA*-pNV*zntA*_2308_com5	∆*zntA* mutant complemented with pNV*zntA*_2308_com5 ^2^	[21]
*B. ovis* ∆*znuA*	∆*znuA* deletion mutant of *B. ovis* PA	This work
*B. ovis* ∆*znuCB*	∆*znuCB* deletion mutant of *B. ovis* PA ^3^	This work
*B. ovis* ∆*znuACB*	∆*znuACB* deletion mutant of *B. ovis* PA ^3^	This work
*B. ovis* ∆*zigA*	∆*zigA* deletion mutant of *B. ovis* PA	This work
*B. ovis* ∆*zur*	∆*zur* deletion mutant of *B. ovis* PA	This work
*B. ovis* ∆*zur*-pBBR1MCS4	∆*zur* mutant bearing pBBR1MCS4	This work
*B. ovis* ∆*zur*-pNV*zur*com2	∆*zur* mutant complemented with pNV*zur*com2 ^2^	This work
*B. ovis* ∆*zigAznuA*	∆*zigAznuA* mutant of *B. ovis* PA ^3^	This work
*B. ovis* ∆*zigAznuACB*	∆*zigAznuACB* mutant of *B. ovis* PA ^3^	This work
*B. ovis* ∆*zntA*∆*zur*	Double ∆*zntA*∆*zur* mutant of *B. ovis* PA ^4^	This work
*B. ovis* ∆*zntA*∆*zur*-pBBR1MCS4	∆*zntA*∆*zur* mutant bearing pBBR1MCS4	This work
*B. ovis* ∆*zntA*∆*zur*-pNV*zntA*_PA_com5	∆*zntA*∆*zur* complemented with pNV*zntA*_PA_com5 ^2^	This work
*B. ovis* ∆*zntA*∆*zur*-pNV*zur*com2	∆*zntA*∆*zur* mutant complemented with pNV*zur*com2 ^2^	This work

^1^ BCCN, Brucella Culture Collection Nouzilly (Institut National de la Recherche Agronomique, Nouzilly, France). ^2^ Multicopy plasmids replicating in *B. ovis* bearing the wild-type gene specified in the plasmid name (*zntA* or *zur* from *B. ovis* PA or *zntA* from *B. abortus* 2308). Plasmids pNV*zntA*_PA_com5 and pNV*zntA*_2308_com5 were previously constructed [21]. ^3^ Single deletion that includes several adjacent genes and intergenic regions (Figure 1). ^4^ Double deletion mutant obtained by deleting *zur* in the ∆*zntA* mutant of *B. ovis* PA obtained previously [21].

## Data Availability

The original contributions presented in this study are included in the article/Supplementary Material. Further inquiries can be directed to the corresponding author.

## Supplementary Materials

The following supporting information can be downloaded at https://www.mdpi.com/article/10.3390/biology15191753/s1, Supplementary Table S1: Primers used in this work; Supplementary Figure S1: Comparison of nt sequences of the entire locus not shown *zigA-zur* of *B. abortus* 2308 and *B. ovis* PA (A), end-point RT-PCR reactions targeting *zigA*, *znuA* or their intergenic region in cDNA obtained from *B. ovis* PA and the Δ*zur* and Δ*znuACB* mutants cultured in TSB-YE-HS for 16 h (B), and picture of the second SDS-PAGE gel used to evaluate the protein profile of selected *B. ovis* mutants and of parental strain cultured in the presence of 35 µM TPEN and 1 or 3 mM ZnCl_2_ (C); Supplementary Figure S2: Growth curves of *B. ovis* PA-pBBR1MCS4, the Δ*zntA*Δ*zur*-pBBRMCS4 double deletion mutant, and the mutant strain complemented with wild-type *zntA* or *zur* genes of *B. ovis* PA in TSB-YE-HS (A) or TSB-YE-HS supplemented with 0.5 mM ZnCl_2_ (B). Growth curves of parental *B. ovis* PA and the Δ*zigA*, Δ*znuA*, Δ*znuCB*, Δ*zur*, Δ*znuACB*, ΔzigA*znuACB*, and Δ*zntA*Δ*zur* mutans in TSB-YE-HS (C) or the same medium supplemented with 5 mM ZnCl_2_ (D), or 0.5 mM EDTA (E); Supplementary Figure S3: Growth curves of *B. ovis* PA and the Δ*zigAznuACB* mutant in the presence of 0.5 mM EDTA or with 0.5 mM EDTA plus 0.5 mM ZnCl_2_ (A), NiCl_2_ (B), MnCl_2_ (C), CoCl_2_ (D), or FeCl_2_ (E), and growth curves of *B. ovis* PA and the Δ*znuACB* (F,G) and ΔzigA*znuACB* (H,I) mutants in the presence of 0.5 mM EDTA or with 0.5 mM EDTA plus 0.5 mM CuCl_2_ (F,H) or MgCl_2_ (G,I); Supplementary Figure S4: Expression of genes related to histidine homeostasis and TonB-mediated transport in the the Δ*zig* and Δ*znuA* mutants of *B. ovis* PA (A) and expression of these genes, as well as genes related to zinc homeostasis, in the Δ*zigAznuA* mutant cultured in the presence of 0.5 mM EDTA (B); Supplementary Data S1: Data for Figures.

## Abbreviations

The following abbreviations are used in this manuscript:

ATPase	Adenosine triphosphatase
cDNA	Complementary DNA
CFU	Colony-forming unit(s)
COG0523	Cluster of Orthologous Groups 0523
DHBA	2,3-dihydroxybenzoic acid
DNA	Deoxyribonucleic acid
EDTA	Ethylenediaminetetraacetic acid
GTP	Guanosine triphosphate
GTPase	Guanosine triphosphatase
HS	Horse serum
iBAQ	Intensity-based absolute quantification
ICP-MS	Inductively coupled plasma mass spectrometry
LC-MS/MS	Liquid chromatography tandem mass spectrometry
LPS	Lipopolysaccharide
MS/MS	Tandem mass spectrometry
OD	Optical density
OM	Outer membrane
PCR	Polymerase chain reaction
RNA	Ribonucleic acid
RQ	Relative quantification
RT-PCR	Reverse transcription polymerase chain reaction
RT-qPCR	Reverse transcription quantitative polymerase chain reaction
SD	Standard deviation
SDS-PAGE	Sodium dodecyl sulfate polyacrylamide gel electrophoresis
TPEN	N,N,N′,N′-Tetrakis(2-pyridylmethyl)ethylenediamine
TSA	Trypticase soy agar
TSB	Trypticase soy broth
YE	Yeast extract

## References

[1] OIE (2021). Chapter 3.8.7. Ovine epididymitis (*Brucella ovis*). Manual of Diagnostic Tests and Vaccines for Terrestrial Animals 2021.

[2] Picard-Hagen N., Berthelot X., Champion J.L., Eon L., Lyazrhi F., Marois M., Peglion M., Schuster A., Trouche C., Garin-Bastuji B. (2015). Contagious epididymitis due to *Brucella ovis:* Relationship between sexual function, serology and bacterial shedding in semen. BMC Vet. Res..

[3] OIE (2021). Chapter 3.1.4. Brucellosis (infection with *B. abortus*, *B. melitensis* and *B. suis*). Manual of Diagnostic Tests and Vaccines for Terrestrial Animals 2021.

[4] Roop R.M., Barton I.S., Hopersberger D., Martin D.W. (2021). Uncovering the Hidden Credentials of *Brucella* Virulence. Microbiol. Mol. Biol. Rev..

[5] Soler-Lloréns P., Gil-Ramírez Y., Zabalza-Baranguá A., Iriarte M., Conde-Álvarez R., Zúñiga-Ripa A., San Román B., Zygmunt M.S., Vizcaíno N., Cloeckaert A. (2014). Mutants in the lipopolysaccharide of *Brucella ovis* are attenuated and protect against *B. ovis* infection in mice. Vet. Res..

[6] Marín C.M., Mainar R.C., de Miguel M.J., Andrés S., Alvarez J.J., Blasco J.M., Muñoz P.M. Re-emergence of *Brucella ovis* infection in Aragon (Spain) after the ban of Rev 1 vaccination. Proceedings of the 72nd Annual Brucellosis Research Conference.

[7] Roop R.M., Gaines J.M., Anderson E.S., Caswell C.C., Martin D.W. (2009). Survival of the fittest: How *Brucella* strains adapt to their intracellular niche in the host. Med. Microbiol. Immunol..

[8] Begg S.L. (2019). The role of metal ions in the virulence and viability of bacterial pathogens. Biochem. Soc. Trans..

[9] Chandrangsu P., Rensing C., Helmann J.D. (2017). Metal homeostasis and resistance in bacteria. Nat. Rev. Microbiol..

[10] Murdoch C.C., Skaar E.P. (2022). Nutritional immunity: The battle for nutrient metals at the host-pathogen interface. Nat. Rev. Microbiol..

[11] Palmer L.D., Skaar E.P. (2016). Transition Metals and Virulence in Bacteria. Annu. Rev. Genet..

[12] Lonergan Z.R., Skaar E.P. (2019). Nutrient Zinc at the Host-Pathogen Interface. Trends Biochem. Sci..

[13] Capdevila D.A., Wang J., Giedroc D.P. (2016). Bacterial Strategies to Maintain Zinc Metallostasis at the Host-Pathogen Interface. J. Biol. Chem..

[14] Braymer J.J., Giedroc D.P. (2014). Recent developments in copper and zinc homeostasis in bacterial pathogens. Curr. Opin. Chem. Biol..

[15] Capdevila D.A., Edmonds K.A., Giedroc D.P. (2017). Metallochaperones and metalloregulation in bacteria. Essays Biochem..

[16] Roop R.M. (2012). Metal acquisition and virulence in *Brucella*. Anim. Health Res. Rev..

[17] Bhubhanil S., Sittipo P., Chaoprasid P., Nookabkaew S., Sukchawalit R., Mongkolsuk S. (2014). Control of zinc homeostasis in *Agrobacterium tumefaciens* via *zur* and the zinc uptake genes *znuABC* and *zinT*. Microbiology.

[18] Schuindt C.P., Batista B.B., Cesetti L., Lima Neto S.P., Soldati T., da Silva Neto J.F. (2026). The genes *zntA* and *zntR* encoding a P-type ATPase and its activator confer zinc and cadmium resistance in *Chromobacterium violaceum*. BioMetals.

[19] Zheng C., Zhai Y., Qiu J., Wang M., Xu Z., Chen X., Zhou X., Jiao X. (2024). ZntA maintains zinc and cadmium homeostasis and promotes oxidative stress resistance and virulence in *Vibrio parahaemolyticus*. Gut Microbes.

[20] Sheehan L.M., Budnick J.A., Roop R.M., Caswell C.C. (2015). Coordinated zinc homeostasis is essential for the wild-type virulence of *Brucella abortus*. J. Bacteriol..

[21] Tartilán-Choya B., Tejedor C., Conde-Álvarez R., Muñoz M.P., Vizcaíno N. (2024). Characterization of three predicted zinc exporters in *Brucella ovis* identifies ZntR-ZntA as a powerful zinc and cadmium efflux system not required for virulence and unveils pathogenic Brucellae heterogeneity in zinc homeostasis. Front. Vet. Sci..

[22] Chaoprasid P., Nookabkaew S., Sukchawalit R., Mongkolsuk S. (2015). Roles of *Agrobacterium tumefaciens* C58 ZntA and ZntB and the transcriptional regulator ZntR in controlling Cd^2+^/Zn^2+^/Co^2+^ resistance and the peroxide stress response. Microbiology.

[23] Kim S., Watanabe K., Shirahata T., Watarai M. (2004). Zinc uptake system (*znuA* locus) of *Brucella abortus* is essential for intracellular survival and virulence in mice. J. Vet. Med. Sci..

[24] Clapp B., Skyberg J.A., Yang X., Thornburg T., Walters N., Pascual D.W. (2011). Protective live oral brucellosis vaccines stimulate Th1 and th17 cell responses. Infect. Immun..

[25] Yang X., Becker T., Walters N., Pascual D.W. (2006). Deletion of *znuA* virulence factor attenuates *Brucella abortus* and confers protection against wild-type challenge. Infect. Immun..

[26] Nairn B.L., Lonergan Z.R., Wang J., Braymer J.J., Zhang Y., Calcutt M.W., Lisher J.P., Gilston B.A., Chazin W.J., de Crecy-Lagard V. (2016). The Response of *Acinetobacter baumannii* to Zinc Starvation. Cell Host Microbe.

[27] Martín-Martín A.I., Sancho P., de Miguel M.J., Fernández-Lago L., Vizcaíno N. (2012). Quorum-sensing and BvrR/BvrS regulation, the type IV secretion system, cyclic glucans, and BacA in the virulence of *Brucella ovis*: Similarities to and differences from smooth brucellae. Infect. Immun..

[28] Kovach M.E., Elzer P.H., Hill D.S., Robertson G.T., Farris M.A., Roop R.M., Peterson K.M. (1995). Four new derivatives of the broad-host-range cloning vector pBBR1MCS, carrying different antibiotic-resistance cassettes. Gene.

[29] Pederick V.G., Eijkelkamp B.A., Begg S.L., Ween M.P., McAllister L.J., Paton J.C., McDevitt C.A. (2015). ZnuA and zinc homeostasis in *Pseudomonas aeruginosa*. Sci. Rep..

[30] Pawlik M.C., Hubert K., Joseph B., Claus H., Schoen C., Vogel U. (2012). The zinc-responsive regulon of *Neisseria meningitidis* comprises 17 genes under control of a Zur element. J. Bacteriol..

[31] Tsolis R.M., Seshadri R., Santos R.L., Sangari F.J., García Lobo J.M., de Jong M.F., Ren Q., Myers G., Brinkac L.M., Nelson W.C. (2009). Genome degradation in *Brucella ovis* corresponds with narrowing of its host range and tissue tropism. PLoS ONE.

[32] Yu N.Y., Wagner J.R., Laird M.R., Melli G., Rey S., Lo R., Dao P., Sahinalp S.C., Ester M., Foster L.J. (2010). PSORTb 3.0: Improved protein subcellular localization prediction with refined localization subcategories and predictive capabilities for all prokaryotes. Bioinformatics.

[33] UniProt C. (2023). UniProt: The Universal Protein Knowledgebase in 2023. Nucleic Acids Res..

[34] Sidhu-Muñoz R.S., Tejedor C., Vizcaíno N. (2020). The Three Flagellar Loci of *Brucella ovis* PA Are Dispensable for Virulence in Cellular Models and Mice. Front. Vet. Sci..

[35] Tartilán-Choya B., Sidhu-Muñoz R.S., Vizcaíno N. (2022). The Transcriptional Regulator MucR, but Not Its Controlled Acid-Activated Chaperone HdeA, Is Essential for Virulence and Modulates Surface Architecture and Properties in *Brucella ovis* PA. Front. Vet. Sci..

[36] Elhassanny A.E., Anderson E.S., Menscher E.A., Roop R.M. (2013). The ferrous iron transporter FtrABCD is required for the virulence of *Brucella abortus* 2308 in mice. Mol. Microbiol..

[37] Yuan J.S., Reed A., Chen F., Stewart C.N. (2006). Statistical analysis of real-time PCR data. BMC Bioinform..

[38] Martín-Martín A.I., Caro-Hernández P., Sancho P., Tejedor C., Cloeckaert A., Fernández-Lago L., Vizcaíno N. (2009). Analysis of the occurrence and distribution of the Omp25/Omp31 family of surface proteins in the six classical *Brucella* species. Vet. Microbiol..

[39] Cox J., Neuhauser N., Michalski A., Scheltema R.A., Olsen J.V., Mann M. (2011). Andromeda: A peptide search engine integrated into the MaxQuant environment. J. Proteome Res..

[40] Cox J., Mann M. (2008). MaxQuant enables high peptide identification rates, individualized p.p.b.-range mass accuracies and proteome-wide protein quantification. Nat. Biotechnol..

[41] Schwanhäusser B., Busse D., Li N., Dittmar G., Schuchhardt J., Wolf J., Chen W., Selbach M. (2011). Global quantification of mammalian gene expression control. Nature.

[42] Sancho P., Tejedor C., Sidhu-Muñoz R.S., Fernández-Lago L., Vizcaíno N. (2014). Evaluation in mice of *Brucella ovis* attenuated mutants for use as live vaccines against *B. ovis* infection. Vet. Res..

[43] Chaoprasid P., Dokpikul T., Johnrod J., Sirirakphaisarn S., Nookabkaew S., Sukchawalit R., Mongkolsuk S. (2016). *Agrobacterium tumefaciens* Zur Regulates the High-Affinity Zinc Uptake System TroCBA and the Putative Metal Chaperone YciC, along with ZinT and ZnuABC, for Survival under Zinc-Limiting Conditions. Appl. Environ. Microbiol..

[44] Edmonds K.A., Jordan M.R., Giedroc D.P. (2021). COG0523 proteins: A functionally diverse family of transition metal-regulated G3E P-loop GTP hydrolases from bacteria to man. Metallomics.

[45] Osterberg M.K., Smith A.K., Campbell C., Deredge D.J., Stemmler T.L., Giedroc D.P. (2024). Coupling of zinc and GTP binding drives G-domain folding in *Acinetobacter baumannii* ZigA. Biophys. J..

[46] Roba A., Carlier E., Godessart P., Naili C., De Bolle X. (2022). Histidine auxotroph mutant is defective for cell separation and allows the identification of crucial factors for cell division in *Brucella abortus*. Mol. Microbiol..

[47] Sieira R., Arocena G.M., Bukata L., Comerci D.J., Ugalde R.A. (2010). Metabolic control of virulence genes in *Brucella abortus*: HutC coordinates *virB* expression and the histidine utilization pathway by direct binding to both promoters. J. Bacteriol..

[48] Mortensen B.L., Rathi S., Chazin W.J., Skaar E.P. (2014). *Acinetobacter baumannii* response to host-mediated zinc limitation requires the transcriptional regulator Zur. J. Bacteriol..

[49] Hood M.I., Mortensen B.L., Moore J.L., Zhang Y., Kehl-Fie T.E., Sugitani N., Chazin W.J., Caprioli R.M., Skaar E.P. (2012). Identification of an *Acinetobacter baumannii* zinc acquisition system that facilitates resistance to calprotectin-mediated zinc sequestration. PLoS Pathog..

[50] Bobrov A.G., Kirillina O., Fosso M.Y., Fetherston J.D., Miller M.C., VanCleave T.T., Burlison J.A., Arnold W.K., Lawrenz M.B., Garneau-Tsodikova S. (2017). Zinc transporters YbtX and ZnuABC are required for the virulence of *Yersinia pestis* in bubonic and pneumonic plague in mice. Metallomics.

[51] Behnsen J., Zhi H., Aron A.T., Subramanian V., Santus W., Lee M.H., Gerner R.R., Petras D., Liu J.Z., Green K.D. (2021). Siderophore-mediated zinc acquisition enhances enterobacterial colonization of the inflamed gut. Nat. Commun..

[52] Bellaire B.H., Elzer P.H., Hagius S., Walker J., Baldwin C.L., Roop R.M. (2003). Genetic organization and iron-responsive regulation of the *Brucella abortus* 2,3-dihydroxybenzoic acid biosynthesis operon, a cluster of genes required for wild-type virulence in pregnant cattle. Infect. Immun..

[53] González Carreró M.I., Sangari F.J., Agüero J., García Lobo J.M. (2002). *Brucella abortus* strain 2308 produces brucebactin, a highly efficient catecholic siderophore. Microbiology.

[54] Vizcaíno N., Pérez-Etayo L., Conde-Álvarez R., Iriarte M., Moriyón I., Zúñiga-Ripa A. (2020). Disruption of pyruvate phosphate dikinase in *Brucella ovis* PA CO_2_-dependent and independent strains generates attenuation in the mouse model. Vet. Res..

[55] Sigdel T.K., Easton J.A., Crowder M.W. (2006). Transcriptional response of *Escherichia coli* to TPEN. J. Bacteriol..

[56] Webster J.M., Bentley M.T., Wojcikiewicz R.J. (2003). N,N,N’,N’-tetrakis(2-pyridylmethyl)ethylenediamine inhibits ligand binding to certain G protein-coupled receptors. Eur. J. Pharmacol..

[57] Noinaj N., Guillier M., Barnard T.J., Buchanan S.K. (2010). TonB-dependent transporters: Regulation, structure, and function. Annu. Rev. Microbiol..

[58] Calmettes C., Ing C., Buckwalter C.M., El Bakkouri M., Chieh-Lin Lai C., Pogoutse A., Gray-Owen S.D., Pomes R., Moraes T.F. (2015). The molecular mechanism of Zinc acquisition by the neisserial outer-membrane transporter ZnuD. Nat. Commun..

[59] Moreno E. (2021). The one hundred year journey of the genus *Brucella* (Meyer and Shaw 1920). FEMS Microbiol. Rev..

[60] Outten C.E., O’Halloran T.V. (2001). Femtomolar sensitivity of metalloregulatory proteins controlling zinc homeostasis. Science.

[61] Outten C.E., Outten F.W., O’Halloran T.V. (1999). DNA distortion mechanism for transcriptional activation by ZntR, a Zn(II)-responsive MerR homologue in *Escherichia coli*. J. Biol. Chem..

[62] Arslan P., Di Virgilio F., Beltrame M., Tsien R.Y., Pozzan T. (1985). Cytosolic Ca^2+^ homeostasis in Ehrlich and Yoshida carcinomas. A new, membrane-permeant chelator of heavy metals reveals that these ascites tumor cell lines have normal cytosolic free Ca^2+^. J. Biol. Chem..

[63] Martín-Martín A.I., Sancho P., Tejedor C., Fernández-Lago L., Vizcaíno N. (2011). Differences in the outer membrane-related properties of the six classical *Brucella* species. Vet. J..

[64] Stranahan L.W., Arenas-Gamboa A.M. (2021). When the Going Gets Rough: The Significance of *Brucella* Lipopolysaccharide Phenotype in Host-Pathogen Interactions. Front. Microbiol..

[65] Silva T.M., Mol J.P., Winter M.G., Atluri V., Xavier M.N., Pires S.F., Paixão T.A., Andrade H.M., Santos R.L., Tsolis R.M. (2014). The predicted ABC transporter AbcEDCBA is required for type IV secretion system expression and lysosomal evasion by *Brucella ovis*. PLoS ONE.

[66] Silva A.P., Macêdo A.A., Costa L.F., Turchetti A.P., Bull V., Pessoa M.S., Araújo M.S., Nascimento E.F., Martins-Filho O.A., Paixão T.A. (2013). *Brucella ovis* lacking a species-specific putative ATP-binding cassette transporter is attenuated but immunogenic in rams. Vet. Microbiol..

[67] Jenner D.C., Dassa E., Whatmore A.M., Atkins H.S. (2009). ATP-Binding Cassette Systems of *Brucella*. Comp. Funct. Genom..

[68] Hou Z., Mitra B. (2003). The metal specificity and selectivity of ZntA from *Escherichia coli* using the acylphosphate intermediate. J. Biol. Chem..

[69] Ammendola S., Cerasi M., Battistoni A. (2014). Deregulation of transition metals homeostasis is a key feature of cadmium toxicity in *Salmonella*. BioMetals.

[70] Martín-Martín A.I., Vizcaíno N., Fernández-Lago L. (2010). Cholesterol, ganglioside GM1 and class A scavenger receptor contribute to infection by *Brucella ovis* and *Brucella canis* in murine macrophages. Microbes Infect..

