# Supplementary material for: Zinc Homeostasis in Brucella ovis Under Zinc Scarcity: Characterization of the ZnuABC Zinc Import System and of a Newly Identified Gene of the Zur Regulon
Source: Biology (Basel). 2026 Oct 2;15(19):1753. doi: 10.3390/biology15191753 (PMC13664203; doi:10.3390/biology15191753)
Supplement: Supplementary file 1 [file biology-15-01753-s001.zip › Supplementary Figure S1.pdf]

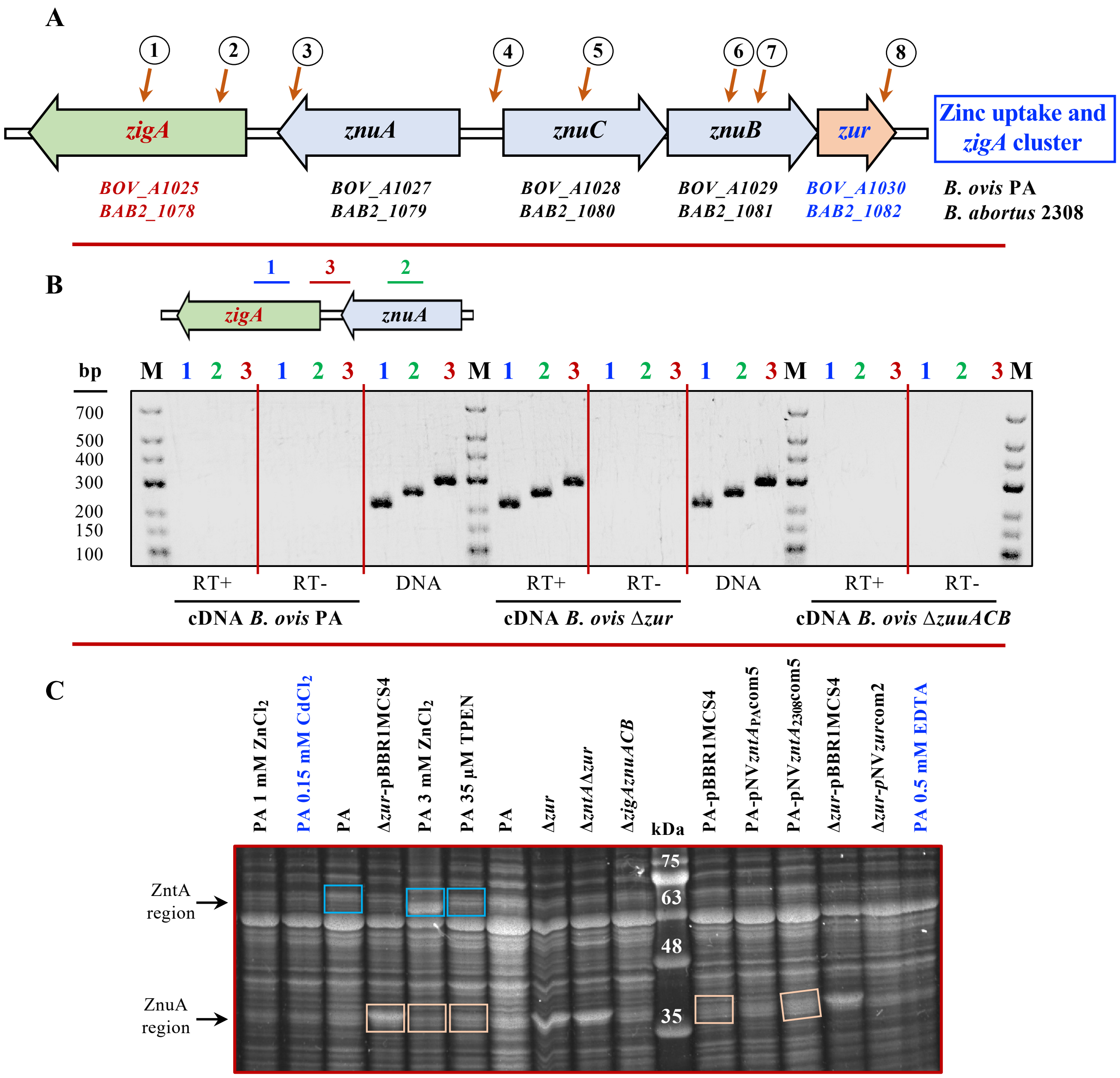

Supplementary Figure S1. Comparison of nt sequences of the entire locus *zigA-zur* of *B. abortus* 2308 and *B. ovis* PA **(A)**, end-point RT-PCR reactions targeting *zigA, znuA* or their intergenic region in cDNA obtained from *B. ovis* PA and the Δ*zur* and Δ*znuACB* mutants cultured in TSB-YE-HS for 16 h **(B)**, and picture of the second SDS-PAGE gel used to evaluate the protein profile of selected *B. ovis* mutants and of parental strain cultured in the presence of 35 µM TPEN and 1 or 3 mM $ZnCl_2$ **(C)**. In panel **(A)**, numbered arrows correspond to the following variants: (1) *zigA*, A>G (Val → Ala); (2) *zigA*, C>A (synonymous; Leu); (3) *znuA*, A>G (Ser → Pro); (4) intergenic region (*znuA-znuC*), C>T (non-coding); (5) *znuC*, A>G (Met → Val); (6) *znuB*, A>G (synonymous; Leu); (7) *znuB*, C>T (Ala → Val); and (8) *zur*, A>G (His → Arg). Locus tags for both species are indicated below each gene. In panel **(B)**, lanes M correspond to the O'GeneRuler Low Range DNA ladder (Thermo Scientific, Vilnius, Lithuania), and regions amplified by end-point RT-PCR (from cDNA resulting from reactions with or without RT, or from DNA) in each lane of the electrophoresis gel are marked with the respective coloured lines in the schematic of the upper part of the panel; primer pairs used were zigAF2-EP+zigART-R (1), znuART-F+znuAR2-EP (2) and zigAF-EP+znuAOVL-R (3). In panel **(C)**, pre-stained protein marker VI (Applichem-Panreac, Barcelona, Spain) is shown and regions analyzed by reversed-phase LC–MS/MS are framed in blue (ZntA region) or orange (ZnuA region). The two samples not shown in Figure 6A are represented in blue.
