# Supplementary material for: Zinc Homeostasis in Brucella ovis Under Zinc Scarcity: Characterization of the ZnuABC Zinc Import System and of a Newly Identified Gene of the Zur Regulon
Source: Biology (Basel). 2026 Oct 2;15(19):1753. doi: 10.3390/biology15191753 (PMC13664203; doi:10.3390/biology15191753)
Supplement: Supplementary file 1 [file biology-15-01753-s001.zip › Supplementary Figure S2.pdf]

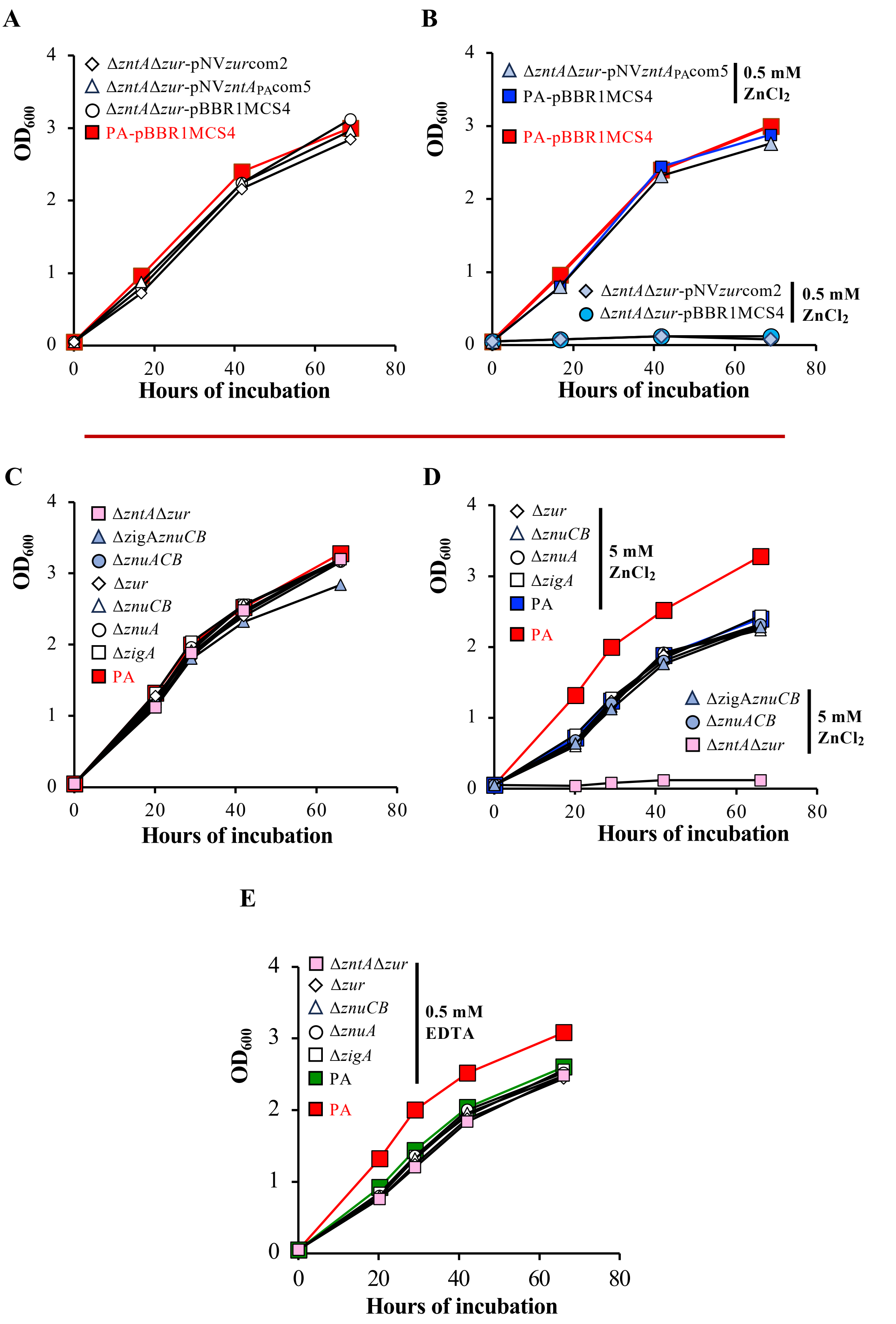

Supplementary Figure S2. Growth curves of *B. ovis* PA-pBBR1MCS4, the Δ*zntA*Δ*zur*-pBBRMCS4 double deletion mutant, and the mutant strain complemented with wild-type *zntA* or *zur* genes of *B. ovis* PA in TSB-YE-HS **(A)** or TSB-YE-HS supplemented with 0.5 mM $ZnCl_2$ **(B).** Growth curves of parental *B. ovis* PA and the Δ*zigA*, Δ*znuA*, Δ*znuCB*, Δ*zur*, Δ*znuACB*, Δzig*AznuACB*, and Δ*zntA*Δ*zur* mutants in TSB-YE-HS **(C)** or the same medium supplemented with 5 mM $ZnCl_2$ **(D)**, or 0.5 mM EDTA (**E**). Growth of the Δ*znuACB*, Δzig*AznuACB* mutants in the presence of 0.5 mM EDTA is shown in Figure 10A.
