# Supplementary material for: Zinc Homeostasis in Brucella ovis Under Zinc Scarcity: Characterization of the ZnuABC Zinc Import System and of a Newly Identified Gene of the Zur Regulon
Source: Biology (Basel). 2026 Oct 2;15(19):1753. doi: 10.3390/biology15191753 (PMC13664203; doi:10.3390/biology15191753)
Supplement: Supplementary file 1 [file biology-15-01753-s001.zip › Supplementary Figure S3.pdf]

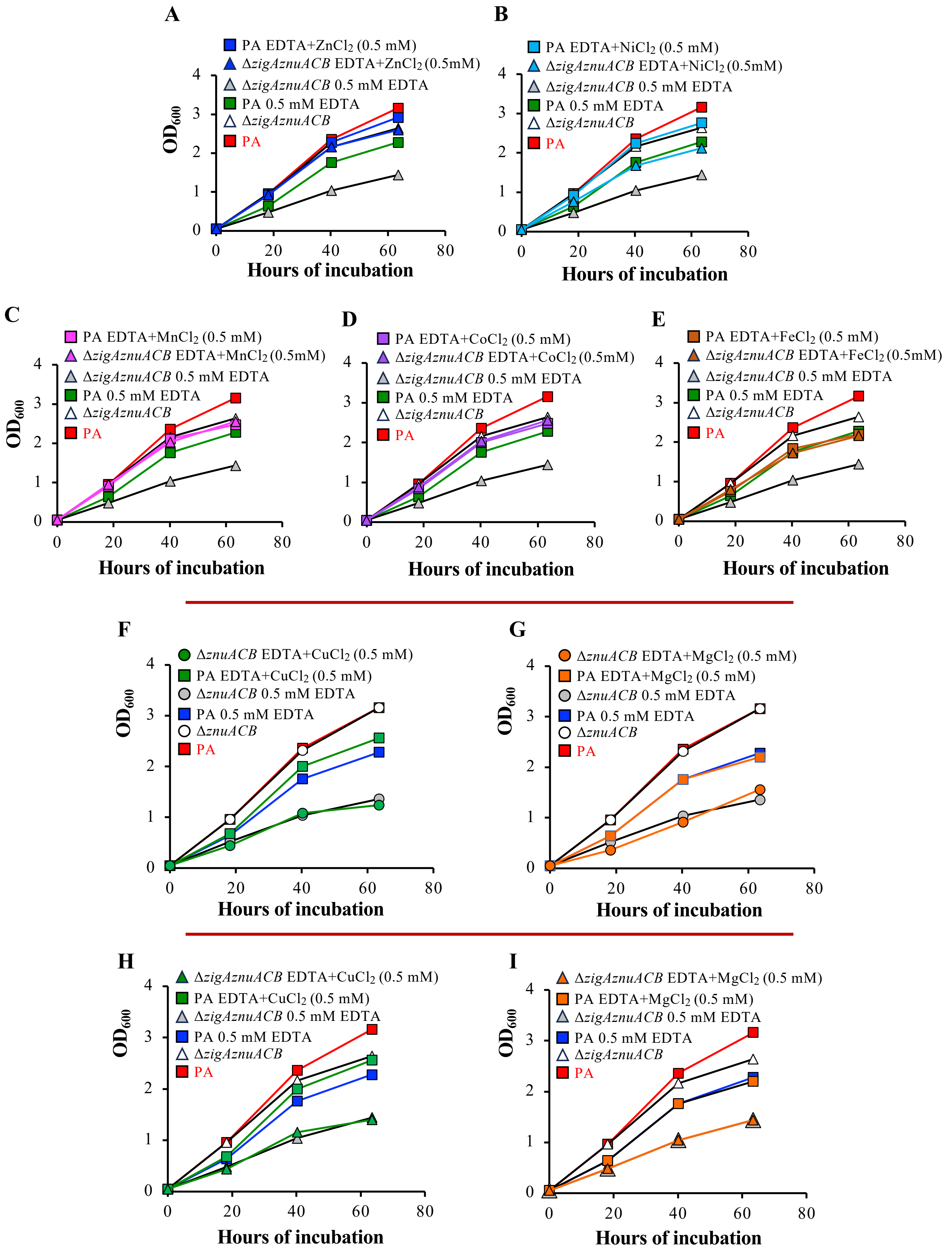

Supplementary Figure S3. Growth curves of *B. ovis* PA and the Δ*zigAznuACB* mutant in the presence of 0.5 mM EDTA or with 0.5 mM EDTA plus 0.5 mM $ZnCl_2$ **(A)**, $NiCl_2$ **(B)**, $MnCl_2$ **(C)**, $CoCl_2$ **(D)**, or $FeCl_2$ **(E)**, and growth curves of *B. ovis* PA and the Δ*znuACB* **(F,G)** and ΔzigA*znuACB* **(H,I)** mutants in the presence of 0.5 mM EDTA or with 0.5 mM EDTA plus 0.5 mM $CuCl_2$ **(F,H)** or $MgCl_2$ **(G,I)**.
