# Supplementary material for: Zinc Homeostasis in Brucella ovis Under Zinc Scarcity: Characterization of the ZnuABC Zinc Import System and of a Newly Identified Gene of the Zur Regulon
Source: Biology (Basel). 2026 Oct 2;15(19):1753. doi: 10.3390/biology15191753 (PMC13664203; doi:10.3390/biology15191753)
Supplement: Supplementary file 1 [file biology-15-01753-s001.zip › Supplementary Figure S4.pdf]

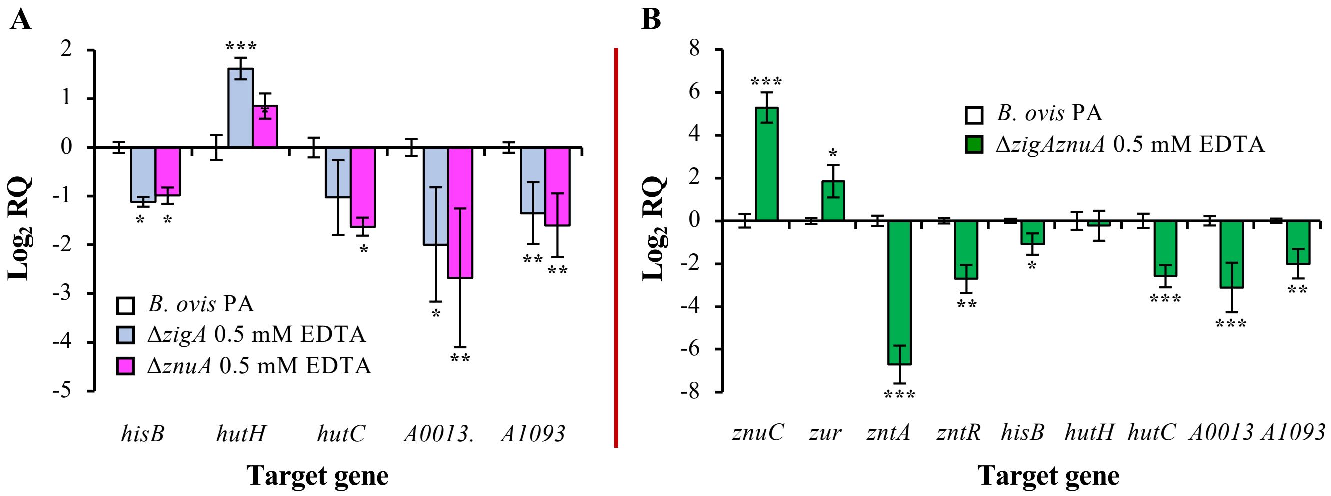

Supplementary Figure S4. Expression of genes related to histidine homeostasis and TonB-mediated transport in the the Δ*zig* and Δ*znuA* mutants of *B. ovis* PA **(A)** and expression of these genes, as well as genes related to zinc homeostasis, in the Δ*zigAznuA* mutant cultured in the presence of 0.5 mM EDTA **(B)**. Statistically significant differences ($p \leq 0.05$) when compared to *B. ovis* PA cultured in normal culture medium are marked with asterisks, *$p \leq 0.05$; **$p \leq 0.01$; ***$p \leq 0.001$.
