# Supplementary material for: Zinc Homeostasis in Brucella ovis Under Zinc Scarcity: Characterization of the ZnuABC Zinc Import System and of a Newly Identified Gene of the Zur Regulon
Source: Biology (Basel). 2026 Oct 2;15(19):1753. doi: 10.3390/biology15191753 (PMC13664203; doi:10.3390/biology15191753)
Supplement: Supplementary file 1 [file biology-15-01753-s001.zip › Supplementary Table S1.pdf]

Supplementary Table S1. Primers used in this work.

Primer name	Nucleotide sequence 5′-3′ [1]	Target gene or plasmid [2]
**Construction of *B. ovis* PA mutants**		
zigAMUT-F [3]	CCGGCATAATTCCATGGT	*zigA (BOV_A1025)*
zigAOVL-R [3]	GGCTTGAGCGATCCATTT	*zigA (BOV_A1025)*
zigAOVL-F	aaatggatcgctcaagccAACGGGAAGCCGCTTTTG	*zigA (BOV_A1025)*
zigAMUT-R	GCCGATATCGCCAAAACG	*zigA (BOV_A1025)*
znuAMUT-F [4]	TCACGCAGAAAATCACCT	*znuA (BOV_A1027)*
znuAOVL-R [4]	CTGAAAGACTGCCTGTCG	*znuA (BOV_A1027)*
znuAOVL-F	cgacaggcagtctttcagAAGAAACAAGGAATGCAG	*znuA (BOV_A1027)*
znuAMUT-R [5]	GTTTCCGCTTTCGCAAGA	*znuA (BOV_A1027)*
zigAznuAOVL-F	aaatggatcgctcaagccAAGAAACAAGGAATGCAG	*zigA* and *znuA*
znuCBMUT-F	ATGTGCGTCATGCTCTTC	*znuCB (BOV_A1028-A1029)*
znuCBOVL-R	GGGGTGAGAGGATTTCTT	*znuCB (BOV_A1028-A1029)*
znuCBOVL-F	aagaaatcctctcaccccTCACCGAAACGGATCGCT	*znuCB (BOV_A1028-A1029)*
znuCBMUT-R [6]	AGATTCACGATCTTGCCG	*znuCB (BOV_A1028-A1029)*
znuACBOVL-F	cgacaggcagtctttcagTCACCGAAACGGATCGCT	*znuACB (BOV_A1027-A1029)*
zigAznuACBOVL-F	aaatggatcgctcaagccTCACCGAAACGGATCGCT	*zigAznuACB (BOV_A1025-A1029)*
zurMUT-F	CTGATGGTCGGCGTGTTT	*zur (BOV_A1030)*
zurOVL-R	GTGCGTCGTCATTGTCCA	*zur (BOV_A1030)*
zurOVL-F	tggacaatgacgacgcacGCATCCCGAAAAGTGTGA	*zur (BOV_A1030)*
zurMUT-R	AAGGTGAGCTTCGAGGAT	*zur (BOV_A1030)*
zurcom-F	TCACCGAAACGGATCGCT	*zur (BOV_A1030)*
**Primers for RT-qPCR and verification of mutant strains [7]**		
16S-RT Fw	TCTCACGACACGAGCTGACG	*16S (BOV_1586)*
16S-RT Rv	CGCAGAACCTTACCAGCCCT	*16S (BOV_1586)*
zntART-F2	GTCAATGGCACGCCTTTT	*zntA (BOV_1942)*
zntART-R2	TGCGACACCTTCCAGCAA	*zntA (BOV_1942)*
zntRRT-F	TTTGTTCATCCGCCATGC	*zntR (BOV_1941)*
zntR-R4	GAAGCTCCACTTCTGTCA	*zntR (BOV_1941)*
zigART-F	TCACGCAGAAAATCACCT	*zigA (BOV_A1025)*
zigART-R	TTCGAGTTTCGCGATGAG	*zigA (BOV_A1025)*
znuART-F	AAGCCCATTGATACGCTG	*znuA (BOV_A1027)*
znuART-R	ATGTGCGTCATGCTCTTC	*znuA (BOV_A1027)*
znuCRT-F2	TTCGCACGATCTGCATCT	*znuC (BOV_A1028)*
znuCRT-R2	ACAGGCGGACATATTCGG	*znuC (BOV_A1028)*
zurRT-F	GCTTTTGCGATCTGCGAA	*zur (BOV_A1030)*
zurRT-R	GCAATTCGAGCGTCGTTT	*zur (BOV_A1030)*
hisBRT-F	TCTGGTCTGGAACGTGAA	*hisB (BOV_2001)*
hisBRT-R	TGGCGCCGTAATGATTGT	*hisB (BOV_2001)*
hutHRT-F	ATGATTGCCGAGGTCACT	*hutH (BOV_A0872)*
hutHRT-R	ATTGCAGGCCATGGAAAC	*hutH (BOV_A0872)*
hutCRT-F	ATACCATCCGTGCCATTG	*hutC (BOV_A0869)*
hutCRT-R	AAGCGTACATGGGTCACA	*hutC (BOV_A0869)*
A0013RT-F	ACGCGGTAGAACATGCCA	*A0013 (BOV_A0013)*
A0013RT-R	AAGGCACGCGAAACTGAT	*A0013 (BOV_A0013)*
A1093RT-F3	GCGTGATTCCGTGAACAT	*A1093 (BOV_A1093)*
A1093RT-R3	TGCCGTTTGTCCTGTTCT	*A1093 (BOV_A1093)*
A0941RT-F	GTCTTTCGCGGCATGTTT	*A0941 (BOV_A0941)*
A0941RT-R	TGACGCTCATCCGGTCAT	*A0941 (BOV_A0941)*
Fe1306RT-F	GTCCTCGGTGAGCGTTAT	*1306 (BOV_1306)*
Fe1306RT-R	TGCCCGCCATTCGTCTTG	*1306 (BOV_1306)*
Fe1612RT-F	CAGGTTGCGCCGATTGAA	*1612 (BOV_1612)*
Fe1612RT-R	AGGTGGCGAGGGAAGCAA	*1612 (BOV_1612)*
**Additional primers used for verification of recombinant plasmids, DNA sequencing or end-point RT-PCR**		
Universal-F	GTTTTCCCAGTCACGAC	Cloning vectors
Universal-R	CAGGAAACAGCTATGAC	Cloning vectors
znuSec-F	TCACCTTTACCGGCAAGA	*znuA (BOV_A1027)*
znuSec-R	CAGCGAATCCGCTGACAA	*znuB (BOV_A1029)*
zigAF2-EP [8]	CAACACCACGACATTGGC	*zigA (BOV_A1025)*
znuAR2-EP [8]	GTCAAGCCAGAAATGCAG	*znuA (BOV_A1027)*
zigAF-EP [8]	TTCCCGATTGTTGAGCAC	*zigA (BOV_A1025)*

[1] Primers were purchased from IDT, Leuven, Belgium. Lowercase sequences in zigAOVL-F, znuAOVL-F, zigAznuAOVL-F, znuCBOVL-F, znuACBOVL-F, zigAznuACBOVL-F and zurOVL-F correspond to regions overlapping with zigAOVL-R, znuAOVL-R, zigAOVL-R, znuCBOVL-R, znuAOVL-R, zigAOVL-R and zurOVL-R respectively. [2] Target gene is the *B. ovis* gene/s to be deleted or PCR-amplified. Primers were designed according to the published genome sequence of *B. ovis* 63/290 (ATCC 25840) (accession numbers NC_009505 and NC_009504 for chromosome I and II, respectively). Primers Universal-F and Universal-R were used for sequencing the DNA insert of the pGEM®-T Easy or pCR®-Blunt recombinant plasmids. [3] Primers used for the construction of the Δ*zigA,* Δ*zigAznuA* and Δ*zigAznuACB* mutants. [4] Primers used for the construction of the Δ*znuA* and Δ*znuACB* mutants. [5] Primer used for the construction of the Δ*znuA* and Δ*zigAznuA* mutants. [3] Primer used for the construction of the Δ*znuCB,* Δ*znuACB* and Δ*zigAznuACB* mutants. [7] Some RT-qPCR primers also served as internal primers for the verification of the mutant strains. [8] End-point RT-PCR primers used together with zigART-R, znuART-F and znuAOVL-R, respectively.
